# Extracting functionally accurate context-specific models of Atlantic salmon metabolism

**DOI:** 10.1038/s41540-023-00280-x

**Published:** 2023-05-27

**Authors:** Håvard Molversmyr, Ove Øyås, Filip Rotnes, Jon Olav Vik

**Affiliations:** 1 Faculty of Chemistry, Biotechnology and Food Science, Norwegian University of Life Sciences, Ås, Norway; 2 Faculty of Biosciences, Norwegian University of Life Sciences, Ås, Norway

**Keywords:** Biochemical networks, Computer modelling

## Abstract

Constraint-based models (CBMs) are used to study metabolic network structure and function in organisms ranging from microbes to multicellular eukaryotes. Published CBMs are usually generic rather than context-specific, meaning that they do not capture differences in reaction activities, which, in turn, determine metabolic capabilities, between cell types, tissues, environments, or other conditions. Only a subset of a CBM’s metabolic reactions and capabilities are likely to be active in any given context, and several methods have therefore been developed to extract context-specific models from generic CBMs through integration of omics data. We tested the ability of six model extraction methods (MEMs) to create functionally accurate context-specific models of Atlantic salmon using a generic CBM (SALARECON) and liver transcriptomics data from contexts differing in water salinity (life stage) and dietary lipids. Three MEMs (iMAT, INIT, and GIMME) outperformed the others in terms of functional accuracy, which we defined as the extracted models’ ability to perform context-specific metabolic tasks inferred directly from the data, and one MEM (GIMME) was faster than the others. Context-specific versions of SALARECON consistently outperformed the generic version, showing that context-specific modeling better captures salmon metabolism. Thus, we demonstrate that results from human studies also hold for a non-mammalian animal and major livestock species.

## Introduction

Within a cell, a multitude of biochemical reactions convert available nutrients into the energy and building blocks required to maintain vital processes and to grow. Metabolism is this vast network of reactions, and the emergence of complete genome sequences and high-throughput experimental technologies has enabled scientists to study it on the system level^1^. Specifically, by identifying and functionally annotating genes in genomes and connecting them to reactions through gene-protein-reaction (GPR) associations, the complete genome-scale metabolic network of an organism can be outlined in a constraint-based model (CBM)^2,3^. The scope and availability of CBMs has increased greatly over the past few decades, largely thanks to databases of metabolic reactions and models^4,5^ and methods for automated reconstruction of microbial metabolic networks from genomes^6^. Today, genome-scale CBMs are readily available for many organisms ranging from microbes to multicellular eukaryotes^7^, and a central goal is to integrate these models with omics data to improve predictions^8,9^.

The mathematics of CBMs are based on the stoichiometric matrix, in which columns represent reactions, rows represent metabolites, and each entry is the stoichiometric coefficient of a metabolite in a reaction. The other key ingredient is a flux vector that represents the rates of all reactions in the network. Because metabolism is much faster than other biological processes with which it interacts, e.g., transcription and translation, metabolite concentrations are assumed to be constant and a steady-state constraint is imposed on the system^10^. Along with bounds and any other linear constraints on fluxes, e.g., reversibility constraints based on thermodynamics, this defines a space of infinitely many solutions, each of which is a feasible combination of fluxes at steady state. This space demarcates an organism’s achievable metabolic states, and thus phenotypes, in a particular environment^11^. Optimal states can be found by optimization methods such as flux balance analysis (FBA)^12^, or the whole solution space can be explored using unbiased methods^13,14^.

A typical CBM contains intracellular biochemical reactions, transport reactions that move metabolites between cellular compartments, and boundary reactions that allow metabolite exchange with the environment. Furthermore, it is common to add a biomass reaction that allows modeling of growth by accounting for the required molecular building blocks and energy. Maximal growth rate, i.e., maximal flux for the biomass reaction, is often used as the primary objective function for FBA, but any linear combination of reaction rates in the model can be minimized or maximized^12^. Parsimonious FBA (pFBA) simply maximizes growth rate before minimizing total flux^15^ but can outperform methods that integrate CBMs with transcriptomics data for microbial flux prediction^16^. On the other hand, it has also been shown that omics integration can help predict fluxes more accurately than pFBA for cancer patients^17^.

Despite the variable performance of transcriptomics-based methods for flux prediction, it is clear that metabolic activities do differ between contexts such as cells or tissues, and these activities ultimately do depend on upstream processes such as gene expression^18^. Generic CBMs, which aim to include all metabolites and reactions found in any cell of an organism, are therefore likely to be superfluous when analyzing specific conditions of interest. Many methods address this by using transcriptomics or other omics data to extract context-specific models rather than to predict or infer fluxes. Context-specific CBMs aim to represent metabolism under a particular set of conditions and have been shown to be more accurate than a generic human CBM^19^.

The utility of context-specific modeling has been demonstrated through several applications. For instance, tissue-specific CBMs extracted from a genome-scale human CBM have been used to study host-pathogen interactions^20^ and brain metabolism^21^ as well as for drug target discovery in cancer^22^. Moreover, context-specific plant CBMs have been used to study fluxes in mesophyll and bundle sheath cells of C_4_ grasses during photosynthesis^23^, the metabolic behavior of organs for production, storage, and consumption of sugars during the generative phase of barley^24^, and stress responses to drought in thale cress^25^. However, context-specific modeling has not been applied to non-mammalian animals such as fish.

Many different model extraction methods (MEMs) have been developed, employing diverse strategies to create context-specific models by reducing a generic CBM^26^. Commonly used MEMs include MBA^27^, mCADRE^28^, FASTCORE^29^, iMAT^30,31^, INIT^32^, and GIMME^33^, which can be categorized into the MBA-like, iMAT-like and GIMME-like families^26^ (Supplementary Table 1). These six MEMs, including their settings, have been thoroughly evaluated for human modeling^19,34,35^, but it has not been clear whether the results translate to other animals for which generic models exist.

In this study, we used the six MEMs with recommended settings to build context-specific models from SALARECON, a generic CBM of Atlantic salmon (*Salmo salar*) metabolism^36^, using liver transcriptomics data contrasting life stages and feeds^37^. We evaluated the context-specific CBMs in terms of their contents and predictions, most importantly their ability to perform metabolic tasks^35,38^, as well as required computation time. Our results show that context-specific model contents and predictions depend heavily on the choice of MEM, but three MEMs produced models that captured significant differences between life stages and one of these was much faster than the others. Context-specific CBMs consistently outperformed SALARECON, supporting context-specific modeling as an approach to explain omics data and improve predictions.

## Results

### Context-specific model contents and predictions

**Fig. 1:**
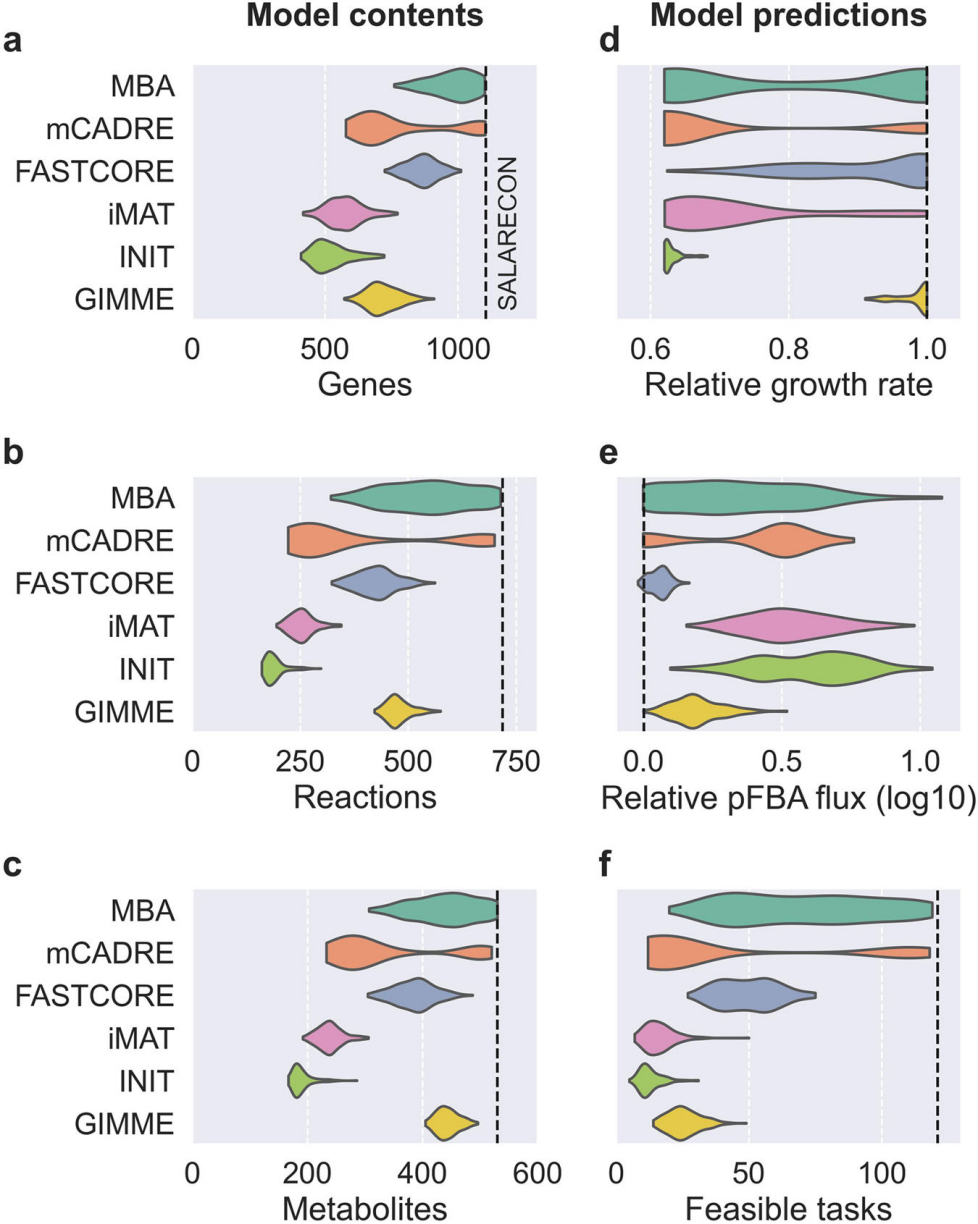
Contents and predictions of context-specific models. Distribution of context-specific model contents (**a**–**c**) and predictions (**d**–**f**) by MEM. **a** Gene counts, **b** reaction counts, **c** metabolite counts, **d** predicted maximal growth rate relative to SALARECON, **e** sum of absolute fluxes from pFBA relative to growth rate, and **f** feasible metabolic task counts. Kernel density estimates are scaled to the same width with cutoffs at the extreme data points. Dashed lines indicate predictions from SALARECON.

We used transcriptomics data from 208 Atlantic salmon liver samples differing in water salinity (life stage) and dietary lipids (feed)^37^. The fish were fed diets containing either fish oil (FO) or vegetable oil (VO) and feed was switched for about half of the fish at each life stage (FO-VO and VO-FO). For each sample and each of the six MEMs, we extracted one context-specific CBM from SALARECON. In total, we extracted 1248 context-specific models, but five mCADRE models were discarded because they were non-functional, leaving 1243 models. The context-specific CBMs varied significantly in their contents as well as in their predictions (Fig. 1). Specifically, models differed between MEMs in terms of gene, reaction, and metabolite counts as well as predicted growth rates, minimal total flux from pFBA^15^, and feasible metabolic tasks^35,38^. Gene, reaction, metabolite, and feasible task counts were highly correlated both between and within MEMs, and larger models tended to predict higher growth rates with less pFBA flux than smaller models (Supplementary Fig. 1). We found no evidence that these statistics were significantly affected by life stage (Supplementary Fig. 2) or feed (Supplementary Fig. 3).

MBA tended not to reduce the generic model as much as other MEMs, keeping most of the 1108 genes, 718 reactions, and 530 metabolites from SALARECON but with comparatively wide distributions for both contents and predictions. Perhaps the most distinguishable MEM was mCADRE, which had bimodal distributions for all contents and predictions: most models were substantially reduced, but some models preserved most of the contents and predictions of SALARECON. Compared to the two other MBA-like MEMs, the contents of FASTCORE models tended to lie between the two mCADRE modes and toward the lower end of the MBA distribution, but FASTCORE predictions were generally more similar to SALARECON. A notable exception is that FASTCORE models performed about half as many metabolic tasks as SALARECON, similar to many MBA and mCADRE models. The two iMAT-like MEMs were very similar across contents and predictions, but iMAT models had a much wider distribution of predicted growth rates than INIT models, all of which had relatively low growth rates. GIMME models were generally most similar to FASTCORE models in their contents, with narrow distributions comparable to the iMAT-like family. Growth rates and minimal flux predictions were close to SALARECON for all GIMME models, but they performed few tasks compared to SALARECON or the MBA-like family.

**Fig. 2:**
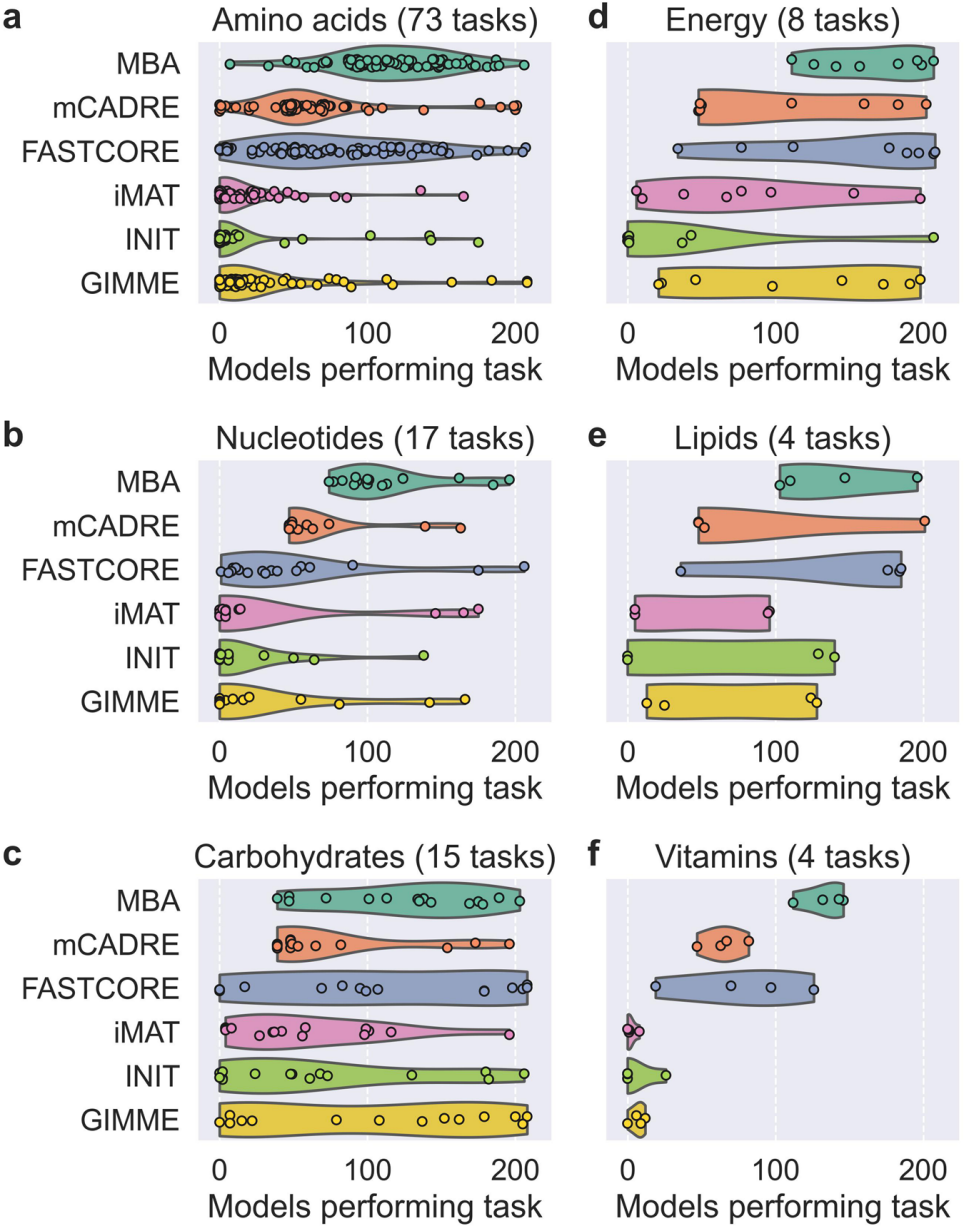
Number of context-specific models performing metabolic tasks. Number of context-specific models in which metabolic tasks were feasible by MEM. Tasks are divided into six metabolic systems: **a** amino acid, **b** nucleotide, **c** carbohydrate, **d** energy, **e** lipid, and **f** vitamin metabolism. Kernel density estimates are scaled to the same width with cutoffs at the extreme data points.

Looking more closely at the number of context-specific models capable of performing each metabolic task, we found clear differences between tasks, MEMs, and metabolic systems (Fig. 2). MBA models tended to perform most of the 121 tasks performed by SALARECON across all systems, reflecting the tendency for MBA to produce models with more reactions than other MEMs. Both MBA and the other MBA-like methods—mCADRE and FASTCORE—had comparatively wide model count distributions, notably spanning the whole range for metabolism of amino acids. The remaining MEMs—iMAT, INIT, and GIMME—were all very similar in terms of the number of models performing tasks. In general, very few models built by these MEMs performed tasks related to metabolism of amino acids, nucleotides, and vitamins, whereas tasks in carbohydrate, energy, and lipid metabolism had a wider range of model counts. The individual tasks that were most frequently performed across all MEMs covered cellular respiration, the thioredoxin system, nucleotide salvage, degradation of ethanol and sugars, as well as synthesis of many amino acids, S-adenosyl methionine (SAM), UDP-glucose, fructose-6-phosphate, glycerol-3-phosphate, and malonyl-CoA (Supplementary Fig. 4). For all MEMs except MBA, there was perfect agreement among models on the feasibility or infeasibility of some tasks. Specifically, all models agreed on two tasks for mCADRE, eight tasks for FASTCORE, 32 tasks for iMAT, 62 tasks for INIT, and 27 tasks for GIMME. We observed no significant effects of life stage (Supplementary Fig. 5) or feed on the number of models performing tasks (Supplementary Fig. 6).

### PCA of reaction presence, task feasibility, and fluxes

**Fig. 3:**
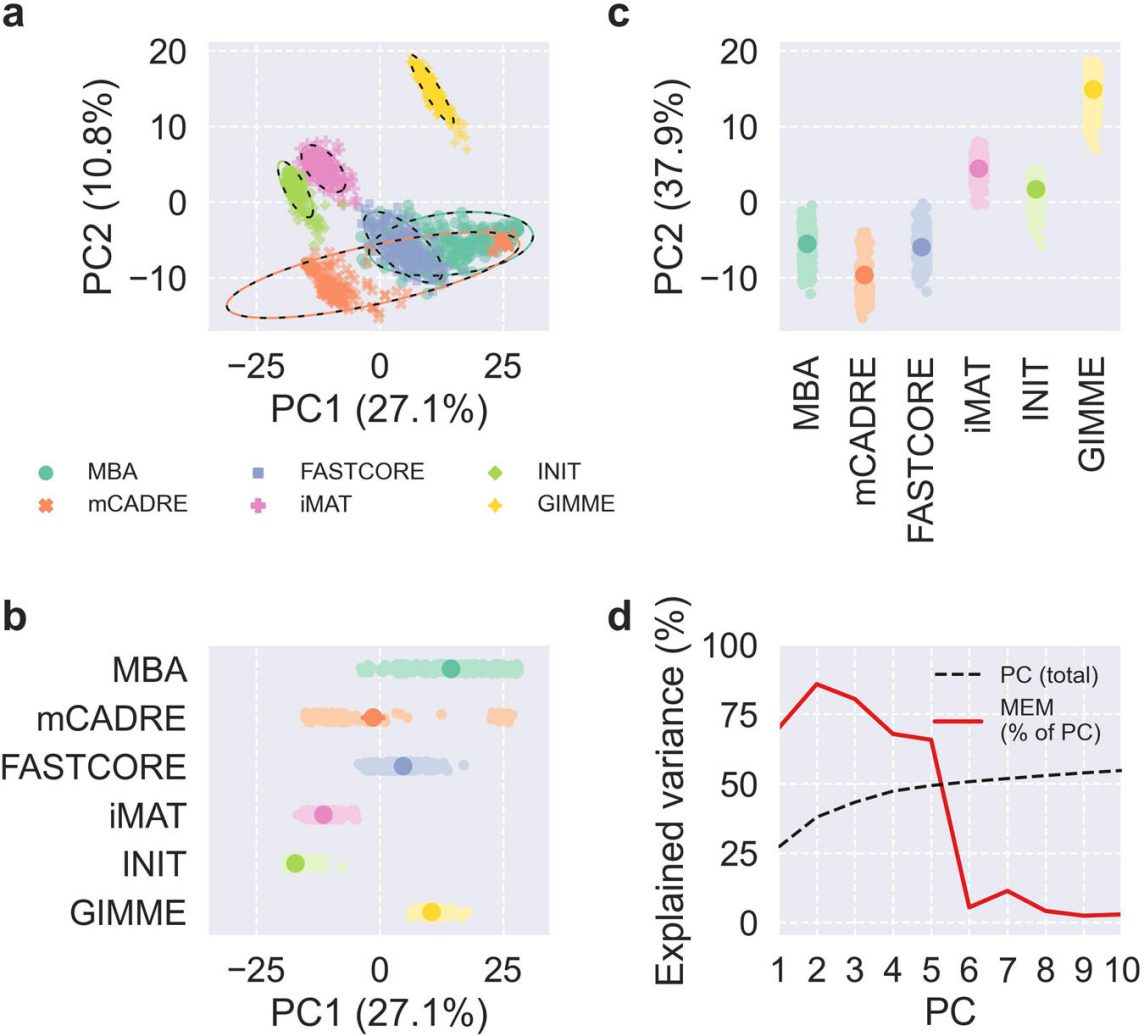
PCA of reaction presence. **a**–**c** Scores of the first two PCs, colored by MEM, with 95% confidence ellipses and intervals. **d** Cumulative total variance explained by the first ten PCs and variance of PC scores explained by MEM.

**Fig. 4:**
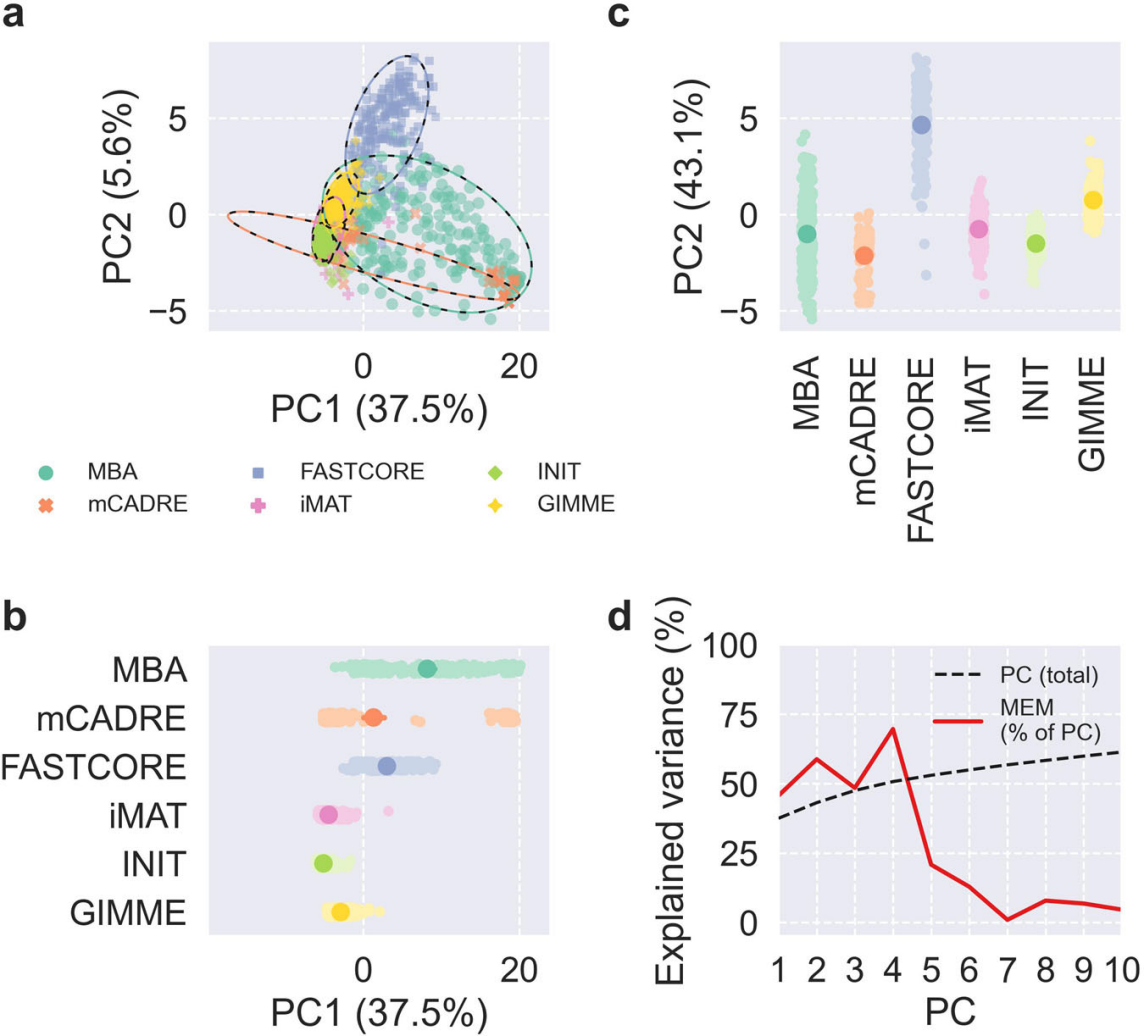
PCA of metabolic task feasibility. **a**–**c** Scores of the first two PCs, colored by MEM, with 95% confidence ellipses and intervals. **d** Cumulative total variance explained by the first ten PCs and variance of PC scores explained by MEM.

To disentangle the contributions of MEM, life stage, and feed to the contents and predictions of the context-specific CBMs, we first applied principal component analysis (PCA) to binary matrices indicating reaction presence (Fig. 3) and metabolic task feasibility (Fig. 4). For both reactions and tasks, the first two principal components (PCs) explained 38% of the total variance for reactions and 43% of the total variance for tasks, and the scores of models were fairly well-separated by MEM in the first two PCs. Also, the first PC primarily explained variability within mCADRE and MBA models, while the second PC explained more variability between MEMs as well as within FASTCORE, iMAT, INIT, and GIMME models. MEM explained most of the variance of the first five PCs for reactions and the first four PCs for tasks, and these PCs explained almost exactly 50% of the total variance. Life stage explained 29% of the variance of the sixth PC from PCA of reactions, but otherwise life stage and feed explained little variance for the first PCs (Supplementary Figs. 7–10). We also applied PCA to the matrix of fluxes predicted by pFBA, producing similar results as seen for reactions and tasks but with less variance explained by MEM and virtually no variance explained by life stage or feed (Supplementary Figs. 11–13). For reactions, tasks, and fluxes, the remaining PCs explained negligible variance with small contributions from MEM, life stage, and feed (Supplementary Fig. 14).

**Fig. 5:**
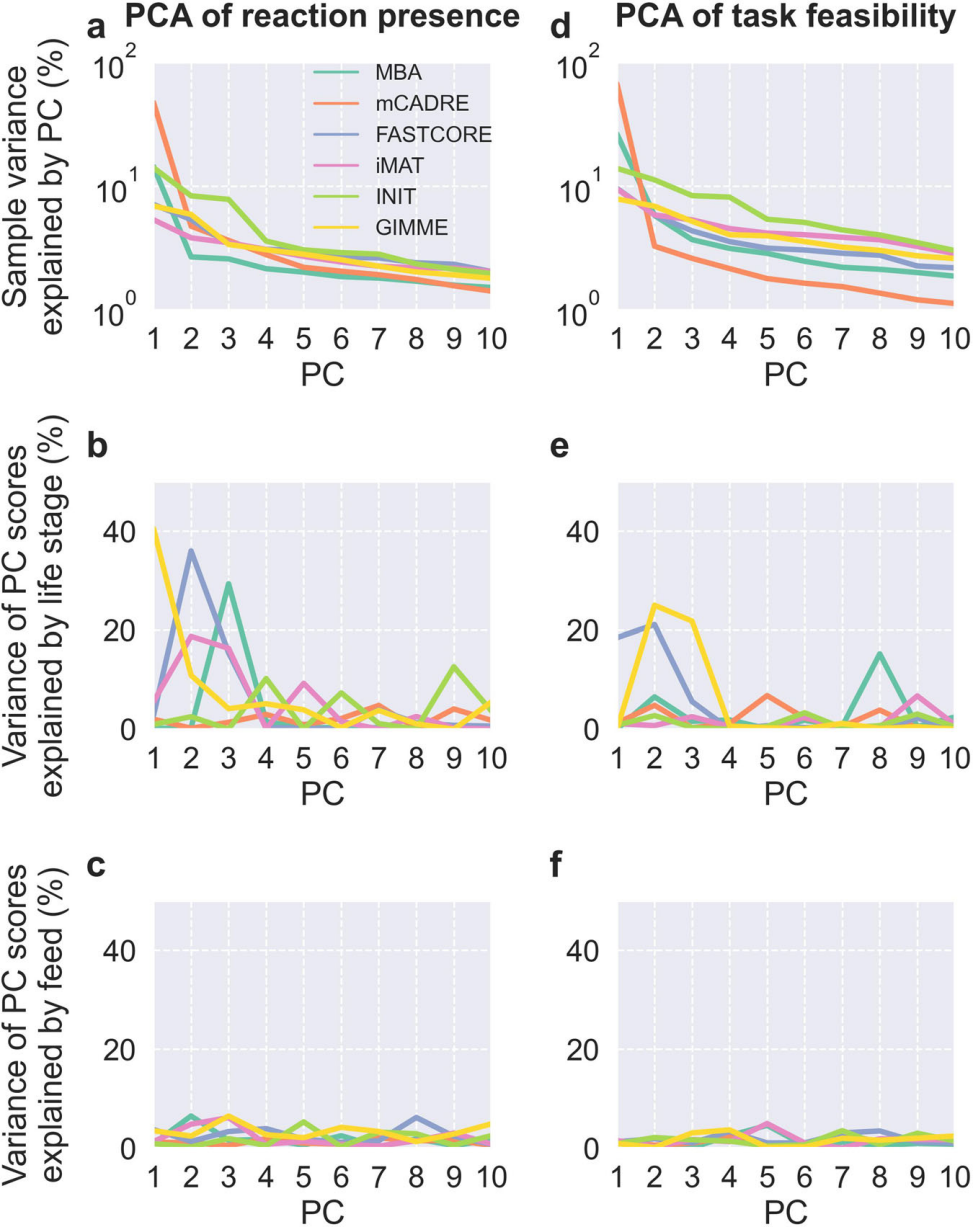
PCA of reaction presence and metabolic task feasibility within each MEM. Results from PCA of reaction presence (**a**–**c**) and task feasibility (**d**–**f**) performed separately for each MEM. Sample variance explained by each PC and variance of PC scores explained by life stage and feed are shown for the first ten PCs.

Hierarchical clustering of the reaction and task matrices recapitulated the results from PCA with clustering mainly by MEM, but it also seemed to reveal further clustering by life stage within at least some MEMs (Supplementary Figs. 15 and 16). We therefore applied PCA to reaction presence and task feasibility within each MEM to further interrogate differences between life stages and feeds (Fig. 5 and Supplementary Figs. 17–40). For both reactions and tasks, the first PC explained more than 10% variance for MBA, mCADRE, and iMAT, and less than 10% for FASTCORE, INIT, and GIMME. The first PC was dominated by outliers for mCADRE and INIT, but INIT’s second PC for tasks also explained more than 10% variance. For three MEMs, life stage explained more than 20% variance for one PC from PCA of reactions: 40% of the first for GIMME, 36% of the second for FASTCORE, and 29% of the third for MBA. For tasks, life stage explained 21% of the second PC for FASTCORE as well as 25% of the second and 22% of the third for GIMME. For pFBA fluxes, we found much less clustering by MEM than for reactions and tasks, but GIMME and FASTCORE models formed fairly distinct clusters (Supplementary Fig. 41). Life stage explained 12% of the variance in both the first and the third PC for PCA within GIMME, which in turn explained 16% and 5% of the total variance, respectively (Supplementary Fig. 42). Feed explained very little variance across all PCs for reactions, tasks, and pFBA fluxes, also within each life stage (Supplementary Figs. 43–45).

### Functional accuracy and computational efficiency

**Fig. 6:**
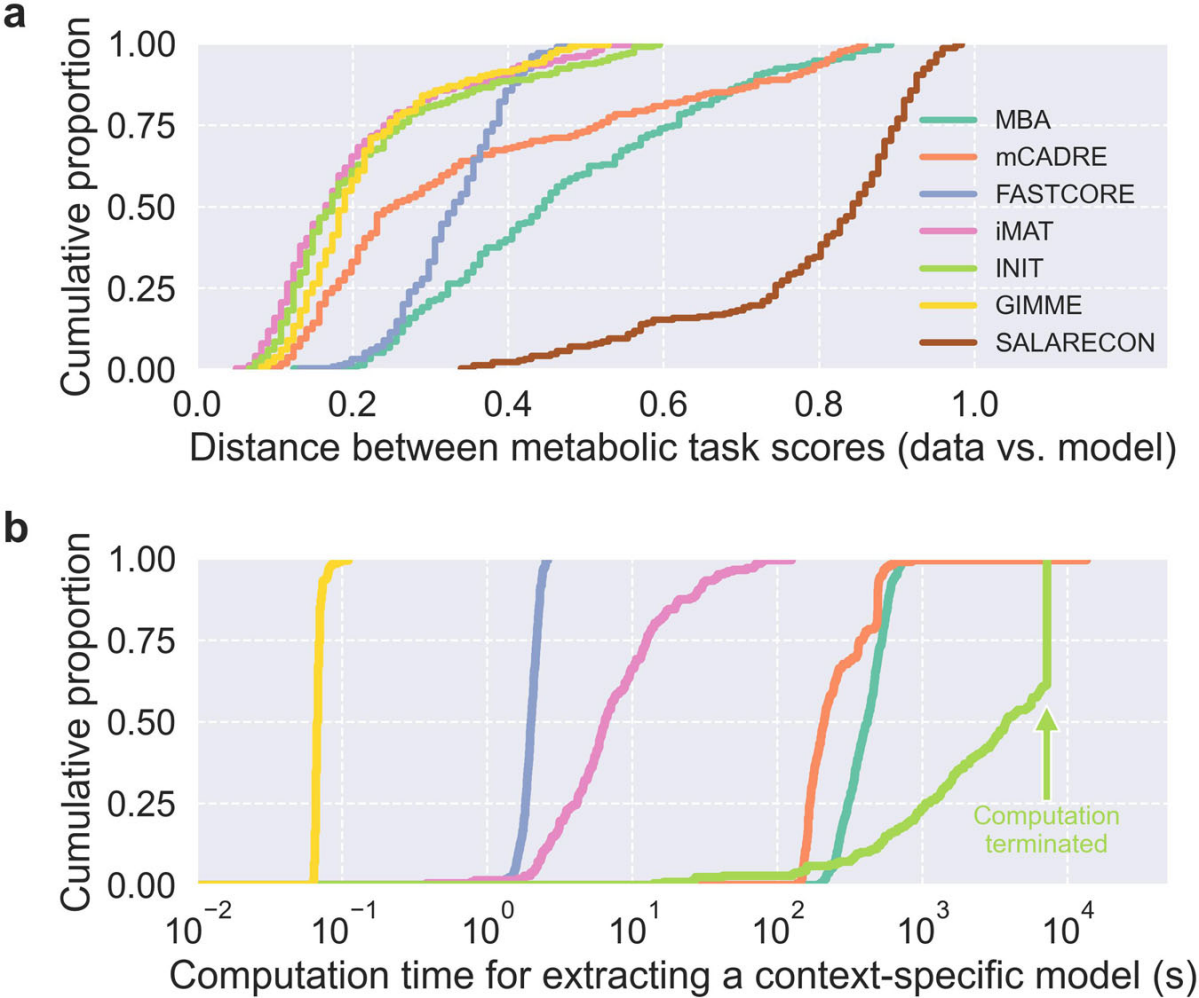
Metabolic task score distance and computation time. Empirical cumulative distributions of (**a**) normalized Hamming distance between binary task scores inferred from data and task feasibility predicted by models, and (**b**) computation time required to extract models.

To evaluate the functional accuracy of the MEMs, we compared metabolic task feasibility predicted by each extracted context-specific CBM to binary task scores inferred directly from the transcriptomics data that was used for extraction^35,38^ (Fig. 6 and Supplementary Fig. 46). Specifically, we computed normalized Hamming distances between task feasibility predicted by the models and task scores inferred from the data. We found that iMAT, INIT, and GIMME outperformed the other MEMs in terms of functional accuracy, meaning that they tended to produce models that predicted task feasibility similar to the inferred task scores. In general, MBA models were the least accurate, with a wide distribution covering larger distances than observed for most iMAT, INIT, and GIMME models. Most models extracted by mCADRE were more accurate than MBA models, albeit with a long tail toward larger distances that likely reflected the bimodality of mCADRE model contents and predictions. FASTCORE models covered the narrowest range of distances and were less accurate than most mCADRE models. Importantly, all MEMs outperformed the baseline defined by SALARECON, meaning that they generally produced models that were more accurate than the generic template model.

The iMAT-like MEMs and GIMME had very similar distributions of distances between predicted and inferred task scores, perhaps with iMAT and INIT outperforming GIMME very slightly. However, looking at distributions of computation time for model extraction, we found that GIMME was by far the most computationally efficient of all the tested MEMs (Fig. 6). All extractions were performed on the same desktop computer (see “Methods”). GIMME was more than an order of magnitude faster than the other MEMs and completed nearly all extractions in less than 0.1 s. FASTCORE was also faster than most MEMs with all extractions finishing within 2.6 s. The remaining MEMs were all less computationally efficient with wider distributions: from 0.4 s to 2 min for iMAT, from 3 min to 14 min for MBA, from 2 min to about 3 h 45 min for mCADRE, and from 14 s to 2 h for INIT, which was generally much slower than the other MEMs.

## Discussion

We found large variation in contents and predictions between MEMs but not between life stages or feeds, which is in line with results from systematic analyses with human models and data^19,35^. Supporting some of our specific findings, one study found that reaction and feasible task counts differed between MEMs largely as we describe and that MEM explained much more variance in PC scores from PCA of task feasibility than tissue or cell type^35^. The only factors affecting context-specific model contents and predictions more than MEM in these studies were rules for applying constraints to the generic template model and thresholds to the transcriptomics data^19,35^. However, we used their recommended constraints and thresholds and therefore did not test these factors (Supplementary Table 1). The fact that results from human studies also hold for a fish demonstrates that MEMs and their settings generalize to other animals than mammals.

MBA and mCADRE clearly differed from the other MEMs in terms of context-specific model contents and predictions. MBA models tended both to be closer to SALARECON and to have wider distributions than models built by other MEMs. This could be at least partially explained by MBA preserving two core reaction sets (high and medium confidence) rather than one, which likely leads to larger models than other MEMs. Notably, the distribution of growth rate predictions from MBA models was bimodal even though all model content distributions were unimodal. Bimodal distributions of predictions were also seen for mCADRE models, but this could be explained by bimodal distributions of contents. However, the causes of the observed bimodality in contents are unclear. Taking all MEMs together, growth rate predictions were bimodal and tended to either be close to the maximum predicted by SALARECON or close to the minimal growth rate requirement that was used for extraction.

FASTCORE and GIMME models preserved the growth rate and pFBA flux of SALARECON to a larger extent than other MEMs. In particular, FASTCORE preserved pFBA flux, likely because it is based on a variation of pFBA^29^, and GIMME preserved growth rate thanks to its default requirement of 90% growth rate relative to the generic model^33^. The iMAT-like MEMs consistently produced the smallest models, which is probably why iMAT and INIT models had low growth rates, high pFBA fluxes, and low feasible task counts. Indeed, this was a tendency across all MEMs: smaller models produced biomass precursors less efficiently and performed fewer metabolic tasks. The most obvious exceptions were GIMME models, which performed fewer tasks than expected based on reaction and metabolite counts, but these counts were in turn larger than expected based on gene counts. The explanation for this is that GIMME, unlike the other MEMs, preserved all exchange reactions, which are not mapped to genes but exchange metabolites with the environment.

There were also notable contrasts between MEMs in terms of the number of models that were able to perform metabolic tasks, reflecting the observed variation in model contents and predictions. For example, tasks in all six metabolic systems tended to be performed by the majority of MBA models, which were the largest, and by many models from the other two MBA-like MEMs—mCADRE and FASTCORE—which also produced comparatively large models. For the iMAT- and GIMME-like MEMs, most tasks were performed by a minority of models and several tasks were infeasible in all models. Perfect agreement between models on task feasibility was more common for iMAT, INIT, and GIMME than for MBA, mCADRE, and FASTCORE. It is important to note that, perhaps counterintuitively, models that perform many tasks are not necessarily better than those that perform few. A good context-specific CBM should ideally perform the tasks that are actually performed by the organism in that context, i.e., tasks for which there is evidence in literature and data.

Despite large variation in task feasibility, some tasks were frequently performed across all MEMs, and we found remarkable agreement between these tasks and metabolic processes known to be important in the liver. For example, consistent feasibility of cellular respiration and sugar degradation is in line with the liver’s role as a hub that transforms dietary nutrients into energy and building blocks for other tissues^39^. However, energy metabolism is fundamental for any cell and its consistent feasibility could simply be driven by growth requirements in the MEMs. Other observations are less likely to be artifacts, e.g., consistent feasibility of the thioredoxin system, which can play an important role in reducing oxidative stress caused by high-fat diets^40^, and nucleotide salvage, which is a key part of the liver’s central control of nucleotide synthesis^41^. The liver also synthesizes many amino acids, including glutamate, glutamine, alanine, aspartate, and glycine, which were most frequently synthesized by context-specific CBMs^42^. Consistent feasibility of SAM synthesis reflects the fact that SAM is essential for liver health and mostly generated in hepatocytes^43^; UDP-glucose is a precursor for glycogen, which is synthesized and stored in fish liver^44^; fructose-6-phosphate synthesis through the pentose phosphate pathway is particularly important in the liver^45^; glycerol-3-phosphate is a precursor for glycerolipids, which are mostly synthesized in the liver^46^; and malonyl-CoA is essential for primarily hepatic fatty acid synthesis^47^.

The liver is a very metabolically active organ, so detecting processes that occur in that organ may not be surprising. However, it is notable that the most frequently performed tasks across MEMs were known to be important for liver metabolism. This supports context-specific modeling as an approach for studying tissue-specific metabolism, and it also suggests ensemble modeling as a potential strategy for managing uncertainty and making context-specific model predictions more robust. Specifically, one could use several different MEMs and template models to build an ensemble of CBMs for each organism and context of interest, i.e., from the same omics data, and predictions could be based on agreement among models in the ensemble^48^. Ensemble modeling could also help improve the models themselves by applying machine learning to their contents and predictions^49^. Indeed, recent studies have demonstrated context-specific ensemble modeling with a single MEM^50^ and combined multiple MEMs to build a single model^51^.

Comparing distributions of model contents and predictions, we saw clear differences between MEMs but not between life stages or feeds. This was unexpected, as we knew that many genes related to metabolism were differentially expressed both between fresh- and saltwater and between FO and VO^37^. To detect contrasts between these factors among context-specific CBMs, we first applied PCA to reaction presence and task feasibility. Results were very similar for reactions and tasks, reflecting the fact that task feasibility is determined by reaction presence. In both cases, the first five PCs explained about half of the total variance in the data, and variance in the scores of these PCs was mainly explained by MEM. Life stage or feed explained comparatively tiny amounts of variance, but hierarchical clustering revealed a tendency for grouping by life stage within MEMs, leading us to perform PCA separately for each MEM. This did indeed lead to PCs capturing differences between life stages—most notably for GIMME, FASTCORE, and MBA—but not between feeds. PCAs within each MEM and life stage also failed to separate models based on diet, possibly due to the simplified representation of lipids in SALARECON. Future studies should expand lipid metabolism in SALARECON to potentially detect differences between feeds. These results show that choice of MEM is by far the most important determinant for model contents and predictions, but at least some of the MEMs are capable of producing models that capture biological differences.

Besides reaction presence and task feasibility, we also applied PCA and hierarchical clustering to pFBA fluxes, revealing less clustering by MEM than for reactions and tasks and hardly any variance explained by life stage or feed. GIMME and FASTCORE models generally clustered together, and GIMME was the only MEM with significant variance in PC scores explained by life stage. Compared to reactions and tasks, pFBA fluxes were more similar across MEMs, reflecting the fact that pFBA predicted a minimal flux distribution that allows maximal growth rate. This means that pFBA fluxes tend to be enriched in core pathways that generate energy and biomass precursors, which are highly conserved across conditions both evolutionarily and by MEMs.

Finally, we evaluated the functional accuracy and computational efficiency of MEMs. Context-specific models should be able to perform metabolic tasks for which there is evidence in the data used for extraction, and we therefore compared predicted task scores to task scores inferred from the data. Even though the same data were used to infer tasks and extract models, task feasibility was not enforced in the extraction process for any of the MEMs. Agreement between inferred and predicted scores tended to be the highest for iMAT, INIT, and GIMME. These were also the MEMs that produced the smallest models, meaning that smaller models tended to be more context-specific than larger ones. FASTCORE models were the most consistent, although they performed slightly worse the iMAT- and GIMME-like models. MBA and mCADRE both had long tails, and some models performed as poorly as SALARECON, the generic template model. GIMME was an order of magnitude faster than FASTCORE, which was in turn faster than iMAT. MBA and mCADRE both required about equally long computation times, while INIT was the slowest, requiring us to terminate the procedure after 2 h. A near-optimal model was always found within this time in a previous study^19^. The differences in efficiency between MEMs are largely as expected based on their optimization strategies: GIMME solves a fixed number of linear programs (LPs), while other MEMs either solve more LPs or much harder mixed-integer linear programs^26^.

There are many other MEMs available that were not systematically tested in this or other studies, including recent methods that account for transcriptomic variability^52^, use ensemble modeling to improve predictions^50^, or combine multiple methods and settings^51^. Notably, some methods integrate metabolic tasks into the model extraction procedure itself by requiring agreement with inferred task feasibility for human models and data^35,53^. This can increase consensus among context-specific CBMs across MEMs^35^ but it does not necessarily improve model contents and predictions relative to simpler methods such as GIMME^17^. There have also been numerous efforts to develop MEMs that integrate multiple types of omics data^54^. In this study, we only focused on transcriptomics data, which is the exclusively accepted data type of most MEMs^54^, and we used metabolic tasks to evaluate the performance of MEMs that have been systematically tested for human applications^19,26,35^. We found that several of the tested MEMs captured expected task feasibility well without enforcing it in the procedure, showing that simple MEMs informed only by transcriptomics can capture key biological differences between contexts. Moreover, all of these MEMs can be extended to integrate tasks^34^ and multi-omics data^54^, so knowing which MEMs likely provide the best baseline will be useful for future studies of context-specific salmon metabolism.

Functionally accurate context-specific models are an important stepping stone toward biotechnological applications. For livestock production, context-specific models can provide a testing ground for optimization of breeding and feed production within constraints that take advantage of conservation laws for more accurate interpretation of omics data^55^. Going further, tissue-specific models could be connected to each other to allow partially dynamic whole-body simulations^56^, which will be necessary to capture energetics and growth at a scale which is meaningful for production biology^57^. Such models will be intermediate in specificity between the generic and sample-specific, and carefully crafted metabolic task lists will be key to their development.

## Methods

### Transcriptomics data and template model

Transcriptomics data and corresponding metadata were downloaded from https://fairdomhub.org/assays/352. The data covered 81,597 transcripts across 208 Atlantic salmon liver samples differing in water salinity (life stage) and dietary lipids (feed). There were 112 samples from the freshwater life stage (pre-smolt) and 96 samples from the saltwater life stage (post-smolt). The fish had been fed diets containing either fish oil (FO) or vegetable oil (VO) with a feed switch for 48 fish at each life stage (FO-VO and VO-FO). A detailed description of the feeding trial and normalization of raw read counts to counts per million (CPM) can be found in the original publication^37^. The most recent version of SALARECON^36^ was downloaded from its GitLab repository (https://gitlab.com/digisal/salarecon) in February 2021. This version contained the same reactions and metabolites as the published version of SALARECON, but it had 29 fewer genes. In total, the model contained 718 reactions, 530 metabolites, and 1104 genes that were mapped to 1109 (14%) of the transcripts. We set the bounds of exchange reactions to allow uptake and secretion of all extracellular metabolites.

### Extracting context-specific models

Six different MEMs were used to extract context-specific models from the transcriptomics data and SALARECON: MBA^27^, mCADRE^28^, FASTCORE^29^, iMAT^30,31^, INIT^32^, and GIMME^33^. We used implementations of these MEMs from the COBRA Toolbox^2^ to extract context-specific models with the function *createTissueSpecificModel*. The implementation of mCADRE did not perform as expected: if removing a reaction led to an infeasible solution, the procedure stopped with an error. This issue was resolved locally and later merged into the COBRA Toolbox (commit 6c1ba69). The parameters needed to execute the different MEMs were set equal to recommended values^19,34,35^ (Supplementary Table 1) where available and default values were used otherwise. As the biomass reaction is not directly associated with any genes, steps were taken to ensure its inclusion in all extracted models. Specifically, its lower flux bound was set to a sufficiently small but otherwise arbitrary value of 1 h^−1^ for all MEMs. This growth rate was not intended to be realistic and only relative growth rates were used in the analyses. For GIMME, we kept the default requirement of preserving 90% of the maximal growth rate of the template model. The biomass reaction was added to the core reaction set of FASTCORE and MBA, assigned a gene score greater than the threshold for GIMME and iMAT, and assigned a specific weight for INIT (Supplementary Table 1). Default flux bounds of ±1,000, depending on reversibility, were used for all other reactions. Model extraction was performed on a Lenovo ThinkStation P340 with an Intel Core i7-10700 (2.9 GHz) processor and 16 GB RAM.

### Context-specific model contents and predictions

For each context-specific model, we counted the number of genes, reactions, and metabolites, computed maximal growth rate with minimal flux using pFBA^15^, and tested the ability to perform metabolic tasks^35,38^. This was done in Python using COBRApy^58^. We obtained a curated and standardized list of 210 metabolic tasks covering seven metabolic systems (amino acid, carbohydrate, energy, lipid, nucleotide, and vitamin and cofactor, and glycan metabolism) from the original publication^35^ and adapted tasks from mammals to salmon by moving metabolites from compartments not included in SALARECON to the cytoplasm and by modifying the expected outcomes of amino acid synthesis tests to match known essentiality^36^. We discarded tasks that could not be performed by SALARECON, leaving 121 tasks that could potentially be performed by context-specific models. We set the bounds of the model to those specified for each task and checked if the task was feasible. Relative growth rate was computed by dividing by the maximal growth rate of SALARECON. We normalized pFBA fluxes by dividing them by maximal growth rate for the extracted model as well as for SALARECON. We then computed relative pFBA flux by dividing the total normalized pFBA flux of the extracted model by the total normalized pFBA flux of SALARECON.

### Gene scores and reaction activity levels

The raw gene expression data was first reduced to only contain genes that were also present in the model. Subsequently, a gene expression threshold was set to determine gene activity in the samples, and any gene with an activity score above this threshold was defined as active. Each gene was given an individual threshold equal to the 90^th^ percentile of its expression value across all samples in the data set, as this has been documented to yield better models than lower thresholds^19^. The 25th percentile of the overall gene expression value distribution (i.e., all genes in all samples) was set as the threshold for any gene with a threshold lower than this percentile. A gene score was then computed for each gene^35,38^:

1 $$\,{{\mbox{Gene score}}}\,=5\ln \left(1+\frac{\,{{\mbox{Expression level}}}}{{{\mbox{Threshold}}}\,}\right)$$

A reaction activity level (RAL) was computed from the gene scores for each reaction in SALARECON through the GPR associations, specifically the maximum expression value amongst all associated genes^35,38^.

### Metabolic task scores

We computed binary metabolic task (MT) scores to determine whether or not tasks were performed in each sample. First, we computed MT scores from the transcriptomics data with reactions and associated genes responsible for executing each task determined by pFBA predictions from SALARECON^15^. These MT scores were calculated as the mean RAL of reactions involved in each task^35,38^:

2 $$\,{{\mbox{MT score}}}\,\,=\frac{\sum \,{{\mbox{RAL}}}}{{{\mbox{Number of reactions involved in the task}}}\,},$$

and made binary by using the recommended threshold of $5\ln (2)$^38^. Second, we used predicted task feasibility as binary MT scores for each context-specific model. For each sample and MEM, we computed the normalized Hamming distance between MT scores inferred from the data and MT scores predicted by the context-specific model.

### Principal component analysis

Principal component analysis (PCA) was performed to assess the impacts of MEM, feed type and life stage on model contents (reactions) and predictions (metabolic task feasibility and pFBA fluxes). We built three matrices in which the rows were models, the columns were either reactions or tasks, and each cell indicated reaction presence, task feasibility, or pFBA flux, respectively. Each column was standardized to have zero mean and unit variance before performing PCA. For each PC, the percentage of variance in PC scores explained by the factors MEM, life stage, and feed was determined by computing the Pearson correlation (*r*) between PC scores and all possible orders of levels for each factor. The maximal *r* across levels was squared to find the explained variance (*R*^2^) for each factor^19^. We performed PCA once for the whole data set, once for each MEM, and once for each life stage within each MEM.

### Reporting summary

Further information on research design is available in the Nature Research Reporting Summary linked to this article.

## Supplementary information

Supplementary information

Reporting Summary

## Data Availability

Data used are available at https://fairdomhub.org/assays/352 and https://gitlab.com/digisal/salarecontext.

## Code availability

Code that reproduces our results is available at https://gitlab.com/digisal/salarecontext.
