# Supplementary material for: Extracting functionally accurate context-specific models of Atlantic salmon metabolism
Source: NPJ Syst Biol Appl. 2023 May 27;9:19. doi: 10.1038/s41540-023-00280-x (PMC10224981; doi:10.1038/s41540-023-00280-x)
Supplement: Supplementary file 1 — Supplementary information [file 41540_2023_280_MOESM1_ESM.pdf]

Supplementary information

Extracting functionally accurate context-specific models of Atlantic salmon metabolism

Håvard Molversmyr[1,2,+], Ove Øyås[1,2,+], Filip Rotnes[1,2], and Jon Olav Vik[1,2,*]

[1]Faculty of Chemistry, Biotechnology and Food Science, Norwegian University of Life Sciences, Ås, Norway
[2]Faculty of Biosciences, Norwegian University of Life Sciences, Ås, Norway
[*]jon.vik@nmbu.no
[+]these authors contributed equally to this work

Constraint-based models (CBMs) are used to study metabolic network structure and function in organisms ranging from microbes to multicellular eukaryotes. Published CBMs are usually generic rather than context-specific, meaning that they do not capture differences in reaction activities, which, in turn, determine metabolic capabilities, between cell types, tissues, environments, or other conditions. Only a subset of a CBM's metabolic reactions and capabilities are likely to be active in any given context, and several methods have therefore been developed to extract context-specific models from generic CBMs through integration of omics data. We tested the ability of six model extraction methods (MEMs) to create functionally accurate context-specific models of Atlantic salmon using a generic CBM (SALARECON) and liver transcriptomics data from contexts differing in water salinity (life stage) and dietary lipids. Three MEMs (iMAT, INIT, and GIMME) outperformed the others in terms of functional accuracy, which we defined as the extracted models' ability to perform context-specific metabolic tasks inferred directly from the data, and one MEM (GIMME) was faster than the others. Context-specific versions of SALARECON consistently outperformed the generic version, showing that context-specific modeling better captures salmon metabolism. Thus, we demonstrate that results from human studies also hold for a non-mammalian animal and major livestock species.

Keywords: Atlantic salmon, systems biology, metabolism, metabolic modeling, data integration, transcriptomics

Supplementary tables

1 2

Supplementary figures

1 3
2 4
3 5
4 6
5 7
6 8
7 9
8 9
9 9
10 9
11 10
12 10
13 10
14 11
15 12
16 13
17 14
18 14
19 14
20 14
21 15
22 15
23 15
24 15
25 16
26 16
27 16
28 16
29 17
30 17
31 17
32 17
33 18
34 18
35 18
36 18
37 19
38 19
39 19
40 19
41 20
42 20
43 21
44 22
45 23
46 23

Supplementary Table 1. Description of the six MEMs, grouped by family, with required inputs as implemented in the COBRA Toolbox and settings used in this study based on recommendations from human studies.

Family	Method	Description	Required inputs	Recommended settings
MBA-like	MBA	Two sets of core reactions are defined with high and medium probability of being active in a given context. MBA then builds a context-specific model containing all high-confidence reactions, as many medium-confidence reactions as possible, and a minimal set of other reactions from the generic CBM to achieve model consistency.	Two sets of core reactions: one with high probability and one with medium probability of being active in the given context.	All reactions associated with a gene score above the 75th percentile of the distribution of all gene scores were added to the high confidence set, while all remaining reactions with a score above $5\ln(2)$ were added to the medium confidence reaction set. The biomass reaction was included in the high-confidence set.
	mCADRE	Using a defined set of core reactions, non-core reactions are pruned based on expression level, connectivity to the core, and a confidence score. Reactions that are not needed to support the core or defined functionalities are removed. If a core reaction is supported by a certain number of unexpressed reactions, it is removed.	Two sets of reaction scores: ubiquity scores quantifying how often each gene is expressed across samples and confidence scores based on evidence from literature.	As the expression distribution of genes is used in the calculation for the gene scores, the gene scores were used as the ubiquity scores. The biomass reaction was given a confidence score of 3. All other reactions were given a score of 1 if they were associated with at least one gene or 0 otherwise.
	FAST-CORE	A set of core reactions that should be active in a certain context of interest is defined, and the algorithm finds a minimal set of reactions that support the core.	Single set of core reactions.	All reactions with a gene score greater than $5\ln(2)$ were added to the core set along with the biomass reaction.
iMAT-like	iMAT	Maximizes the number of matches between a reaction's minimal flux and the group it belongs to, i.e., highly or lowly expressed. Thus, it finds a trade-off between including highly expressed reactions and removing lowly expressed reactions.	Two thresholds defining unexpressed and expressed genes along with the gene expression values.	Gene scores were used as expression values and the upper and lower thresholds were both set to $5\ln(2)$. The biomass reaction was assigned a score of $10\ln(2)$.
	INIT	Finds an optimal trade-off between including and removing reactions based on weights.	A weight for each reaction, which is positive or negative for highly or lowly expressed reactions, respectively.	Reactions with gene score below $5\ln(2)$ were given a weight of -8. Gene score divided by $5\ln(2)$ was used as weight for other reactions. The maximum weight was used for the biomass reaction.
GIMME-like	GIMME	Removes reactions associated with an expression level below a user-defined threshold. Subsequently, reactions are reinserted to achieve a required metabolic function.	A gene expression dataset and an objective function.	Gene scores were used as expression values and the upper and lower thresholds were both set to $5\ln(2)$. The biomass reaction was assigned a score of $10\ln(2)$.

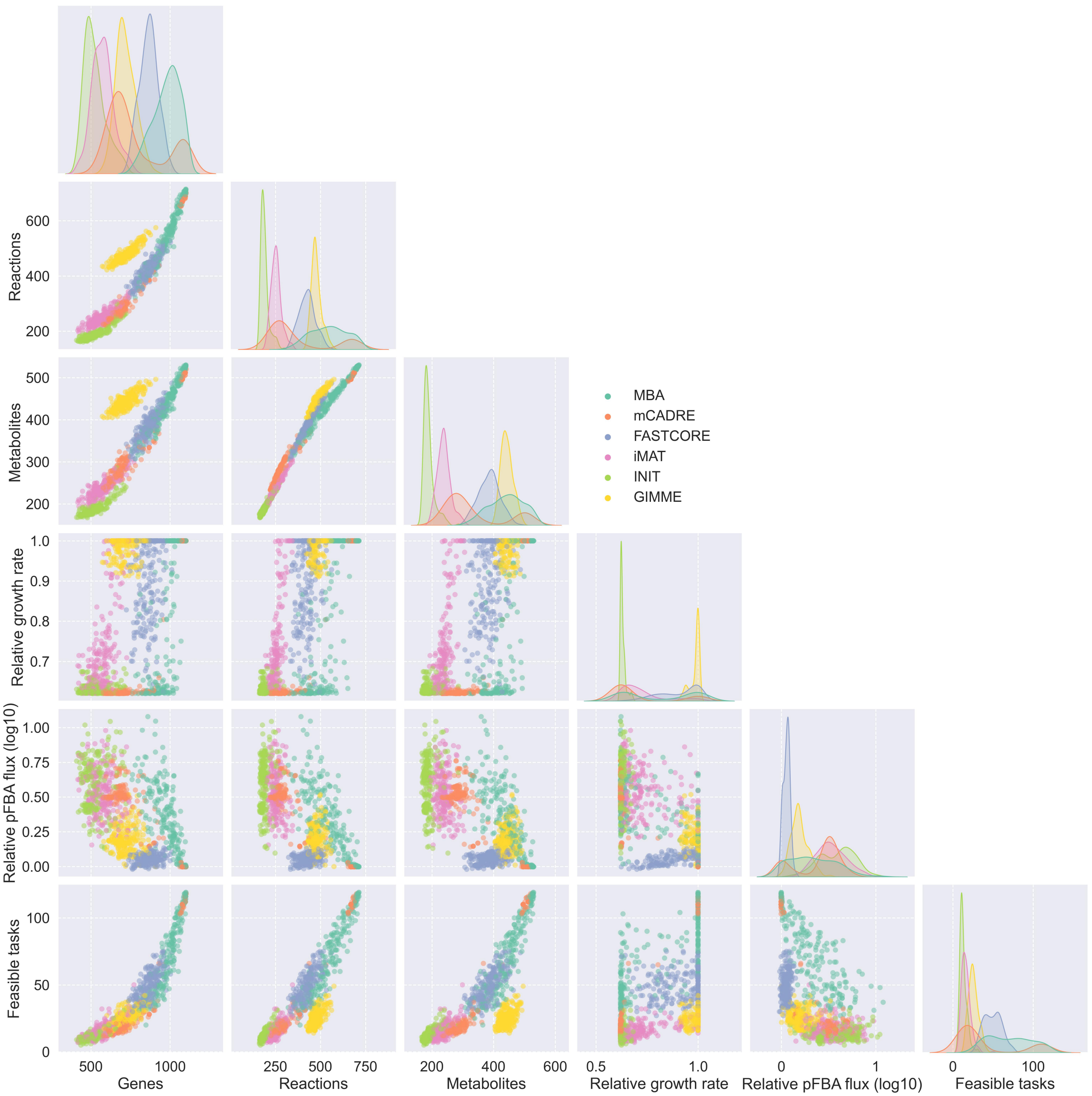

Supplementary Figure 1. Pairwise relationships between context-specific model contents and predictions by MEM. Gene counts, reaction counts, metabolite counts, predicted maximal growth rate relative to SALARECON, sum of absolute fluxes from pFBA relative to growth rate, and feasible metabolic task counts are shown.

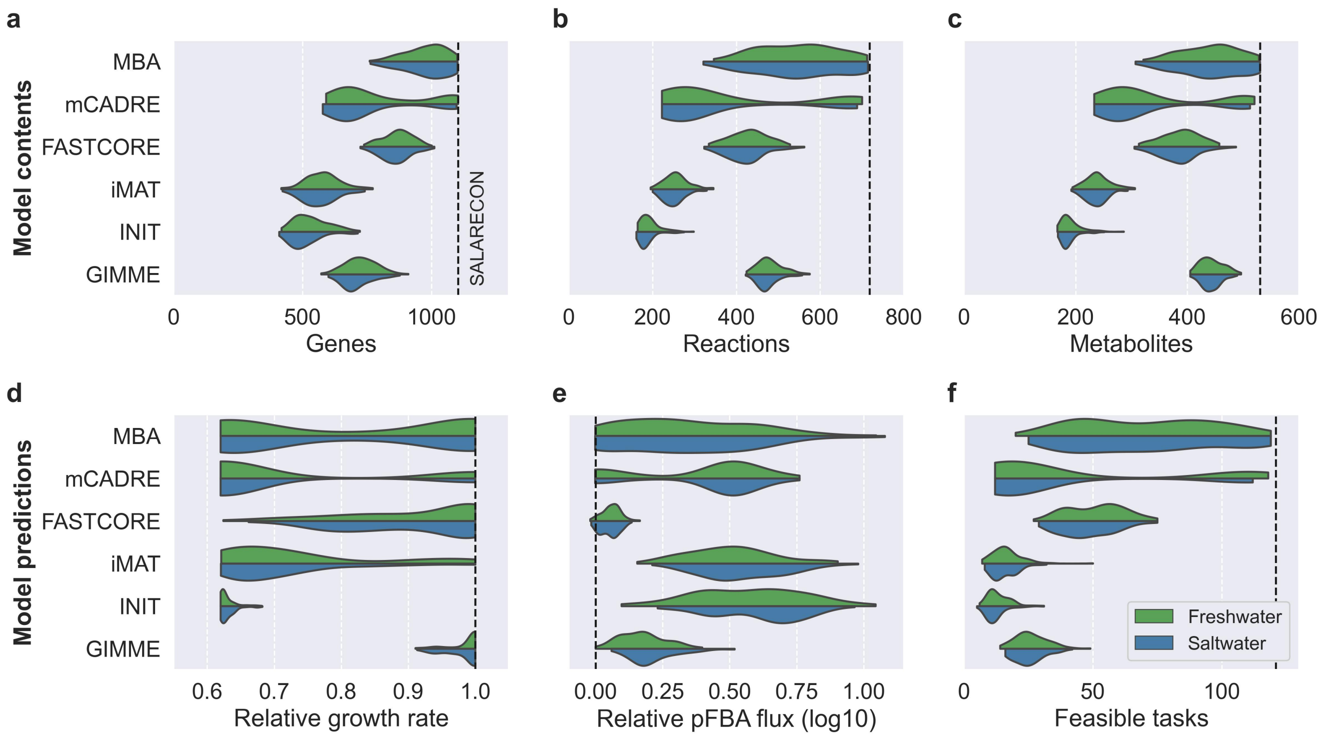

Supplementary Figure 2. Distribution of context-specific model contents (**a–c**) and predictions (**d–f**) by MEM and life stage. (**a**) Gene counts, (**b**) reaction counts, (**c**) metabolite counts, (**d**) predicted maximal growth rate relative to SALARECON, (**e**) sum of absolute fluxes from pFBA relative to growth rate, and (**f**) feasible metabolic task counts. Kernel density estimates are scaled to the same width with cutoffs at the extreme data points. Dashed lines indicate predictions from SALARECON.

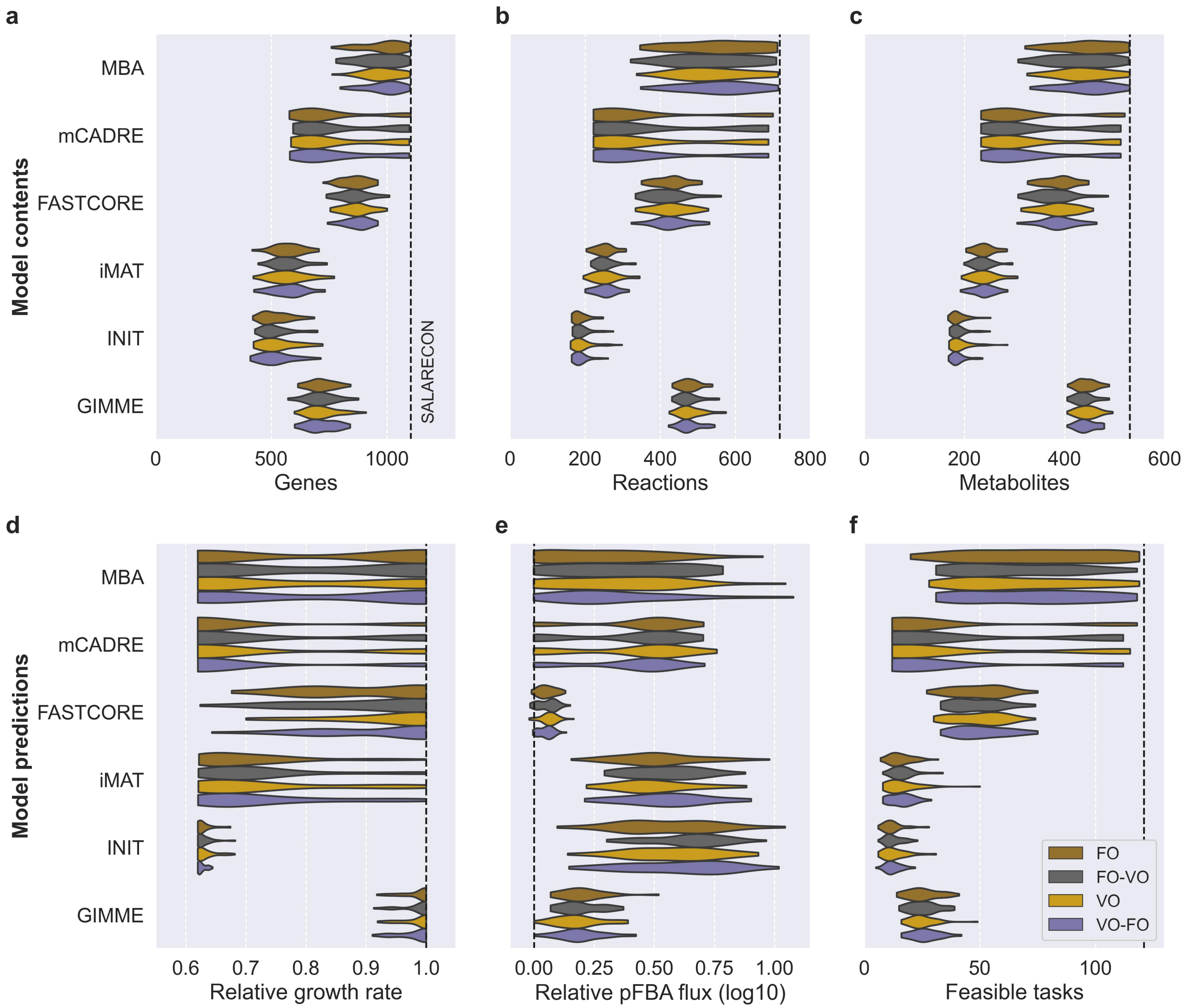

Supplementary Figure 3. Distribution of context-specific model contents (**a–c**) and predictions (**d–f**) by MEM and diet. (**a**) Gene counts, (**b**) reaction counts, (**c**) metabolite counts, (**d**) predicted maximal growth rate relative to SALARECON, (**e**) sum of absolute fluxes from pFBA relative to growth rate, and (**f**) feasible metabolic task counts. Kernel density estimates are scaled to the same width with cutoffs at the extreme data points. Dashed lines indicate predictions from SALARECON.

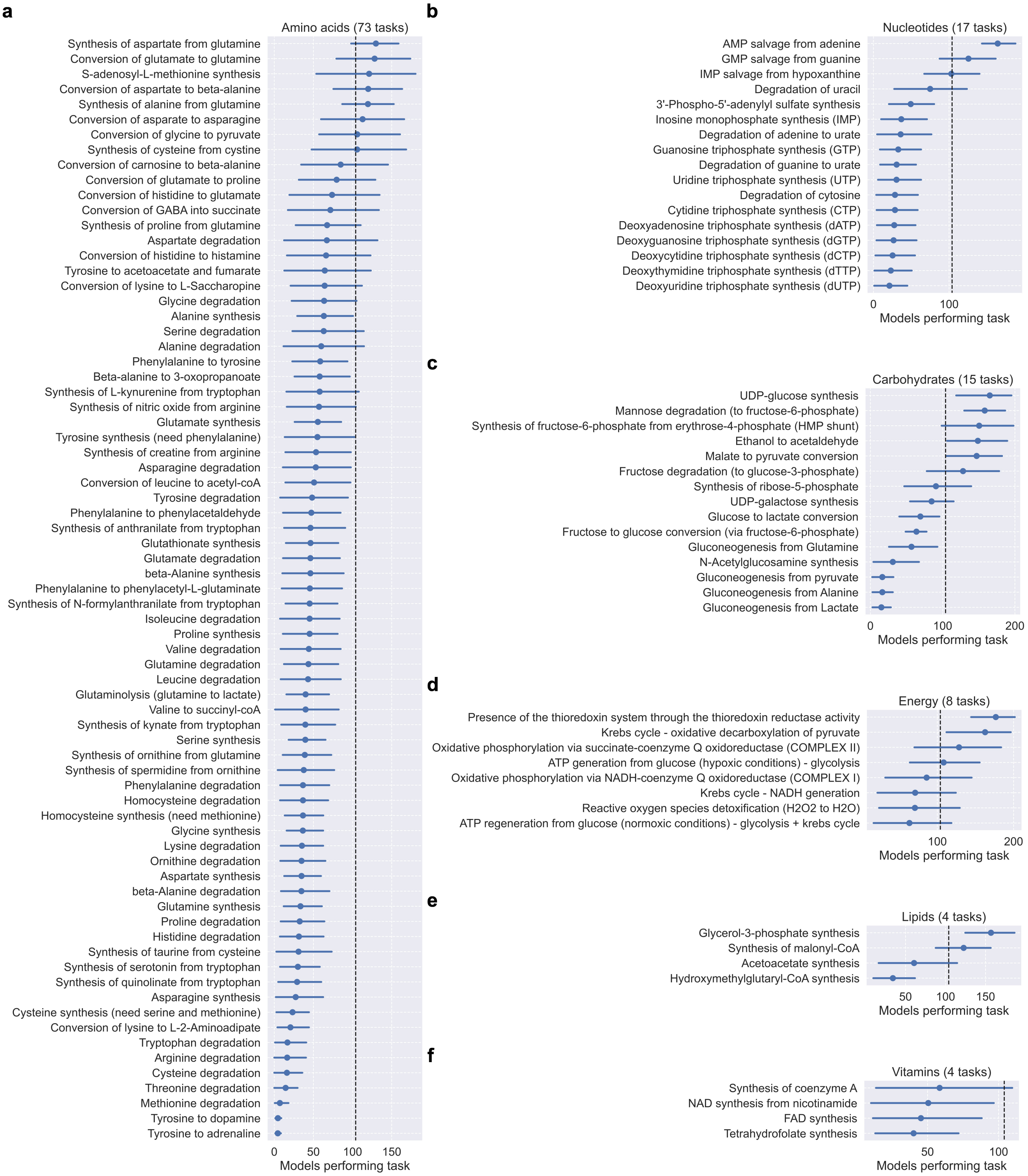

Supplementary Figure 4. Mean number of context-specific models performing each metabolic task Mean number of context-specific models in which each metabolic task is feasible across MEMs. Tasks are divided into six metabolic systems: (**a**) amino acid, (**b**) nucleotide, (**c**) carbohydrate, (**d**) energy, (**e**) lipid, and (**f**) vitamin metabolism. Error bars indicate 95% confidence intervals for the estimated means obtained from bootstrapping with 1,000 samples. Dashed lines indicate half the number of models extracted with each MEM.

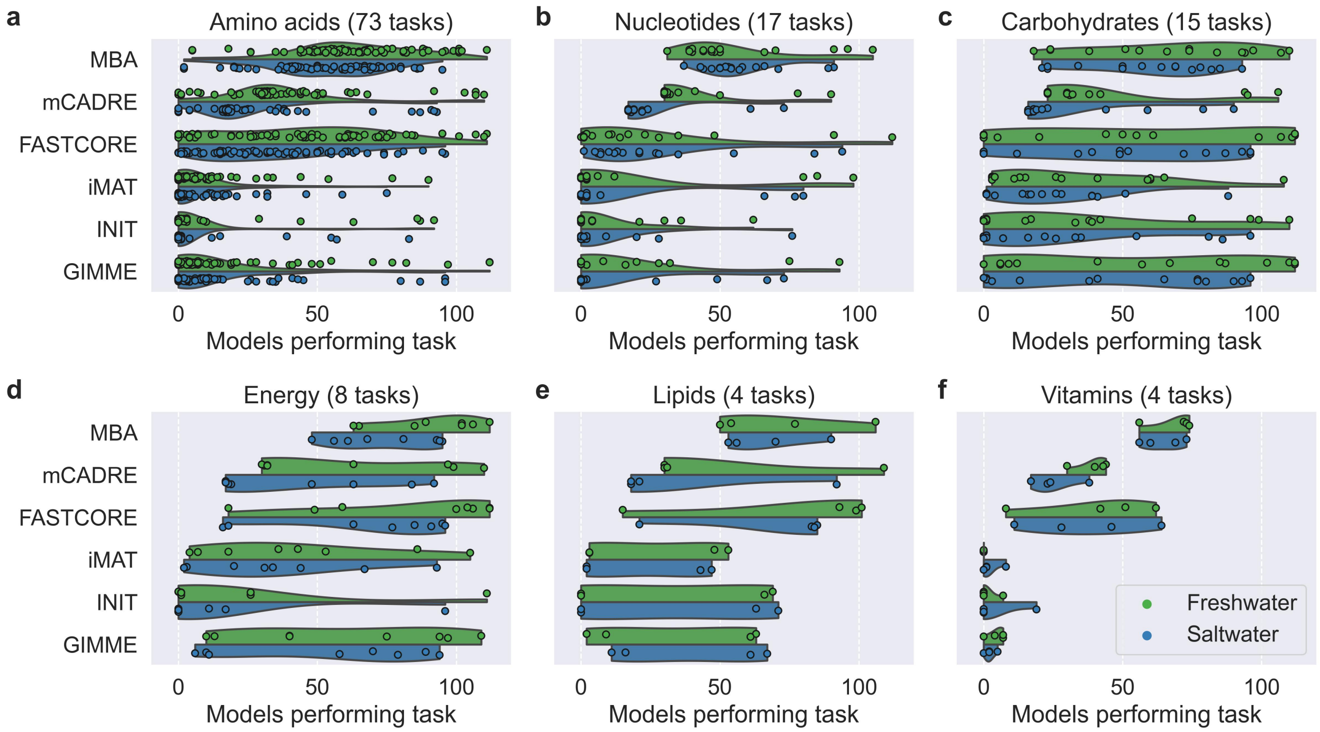

Supplementary Figure 5. Number of context-specific models in which metabolic tasks were feasible by MEM and life stage. Tasks are divided into six metabolic systems: (**a**) amino acid, (**b**) nucleotide, (**c**) carbohydrate, (**d**) energy, (**e**) lipid, and (**f**) vitamin metabolism. Kernel density estimates are scaled to the same width with cutoffs at the extreme data points.

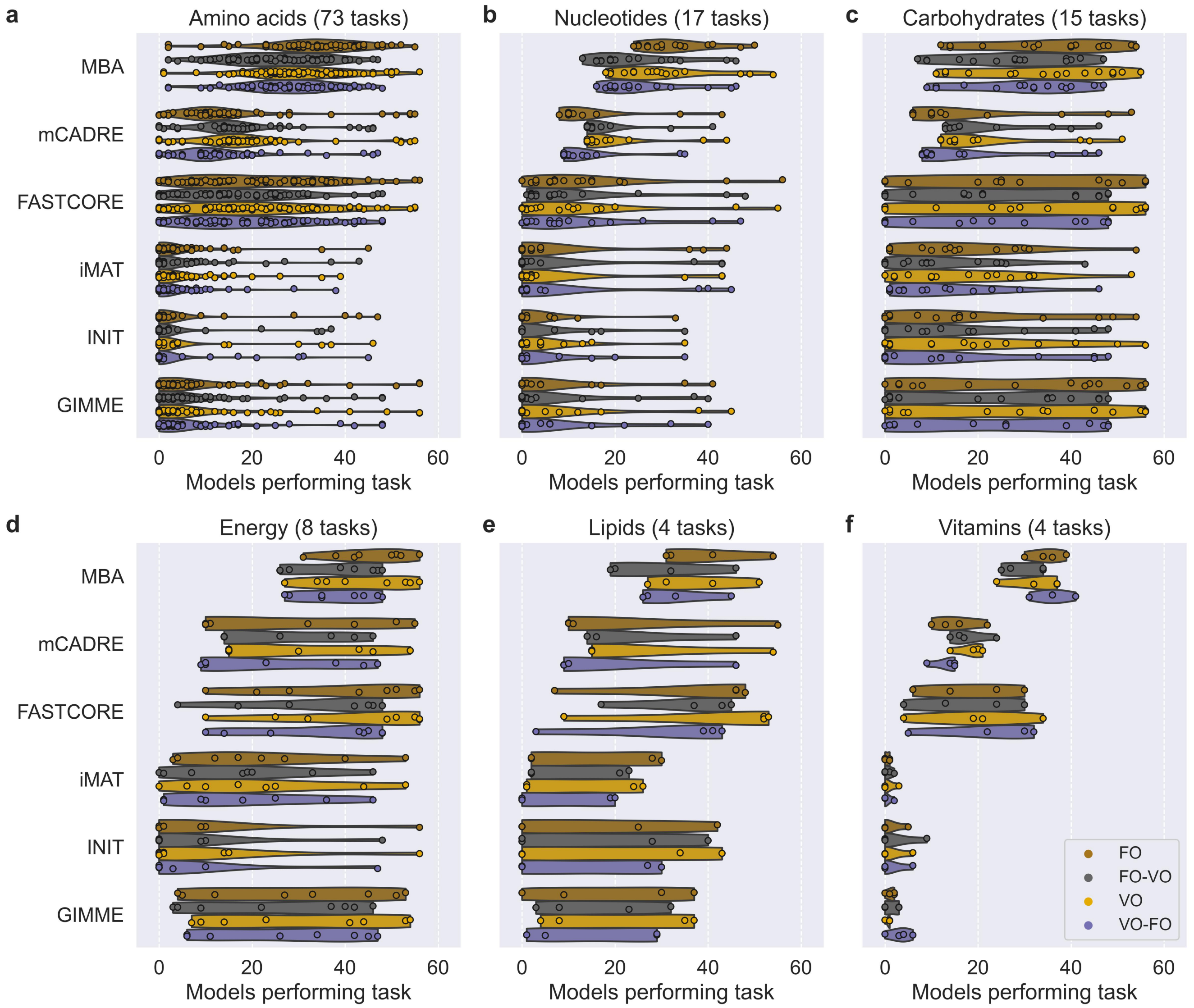

Supplementary Figure 6. Number of context-specific models in which metabolic tasks were feasible by MEM and feed. Tasks are divided into six metabolic systems: (**a**) amino acid, (**b**) nucleotide, (**c**) carbohydrate, (**d**) energy, (**e**) lipid, and (**f**) vitamin metabolism. Kernel density estimates are scaled to the same width with cutoffs at the extreme data points.

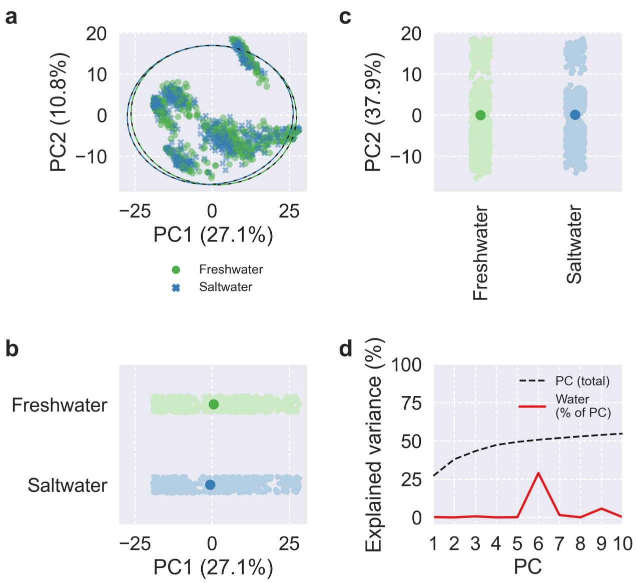

Supplementary Figure 7. Scores and explained variance from PCA of reaction presence. (**a–c**) Scores of the first two PCs, colored by life stage, with 95% confidence ellipses and intervals. (**d**) Cumulative total variance explained by the first ten PCs and variance of PC scores explained by life stage.

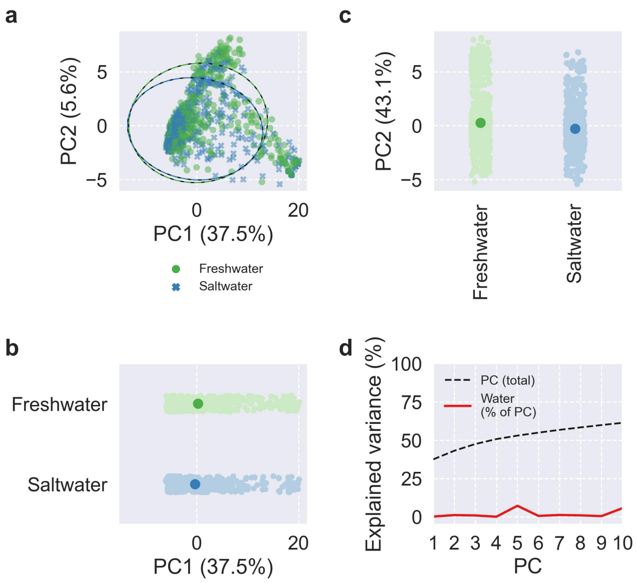

Supplementary Figure 8. Scores and explained variance from PCA of task feasibility. (**a–c**) Scores of the first two PCs, colored by life stage, with 95% confidence ellipses and intervals. (**d**) Cumulative total variance explained by the first ten PCs and variance of PC scores explained by life stage.

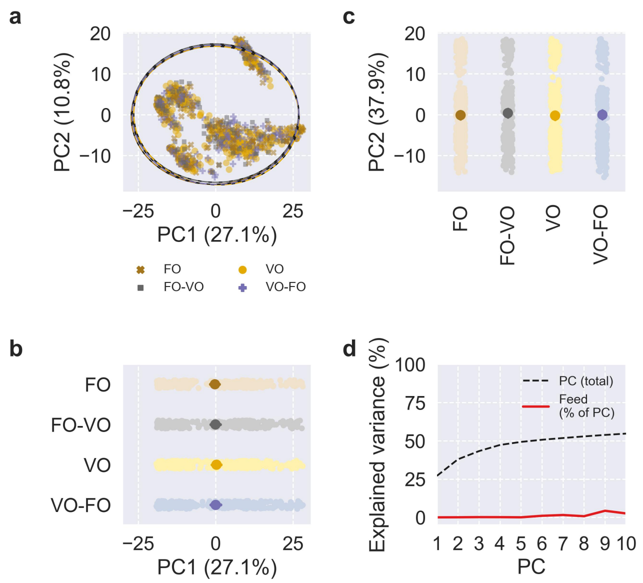

Supplementary Figure 9. Scores and explained variance from PCA of reaction presence. (**a–c**) Scores of the first two PCs, colored by feed, with 95% confidence ellipses and intervals. (**d**) Cumulative total variance explained by the first ten PCs and variance of PC scores explained by feed.

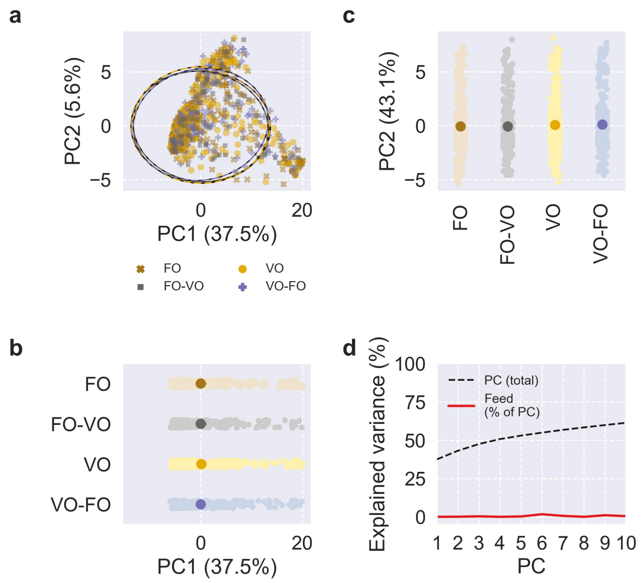

Supplementary Figure 10. Scores and explained variance from PCA of task feasibility. (**a–c**) Scores of the first two PCs, colored by feed, with 95% confidence ellipses and intervals. (**d**) Cumulative total variance explained by the first ten PCs and variance of PC scores explained by feed.

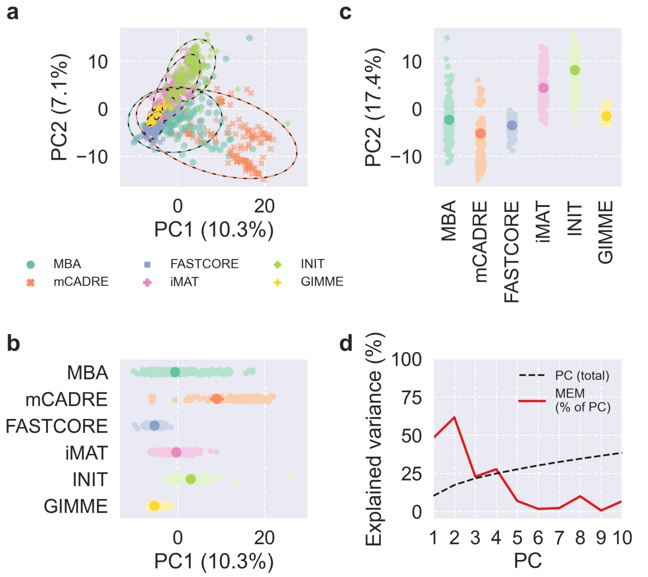

Supplementary Figure 11. Scores and explained variance from PCA of pFBA fluxes. (**a–c**) Scores of the first two PCs, colored by MEM, with 95% confidence ellipses and intervals. (**d**) Cumulative total variance explained by the first ten PCs and variance of PC scores explained by MEM.

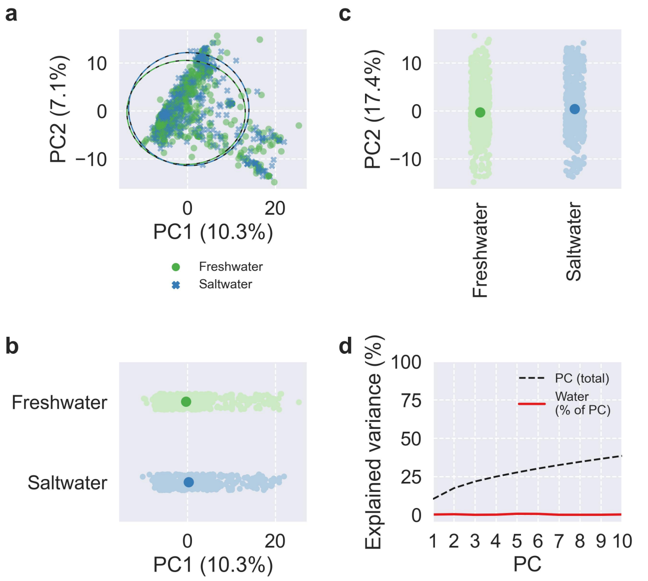

Supplementary Figure 12. Scores and explained variance from PCA of pFBA fluxes. (**a–c**) Scores of the first two PCs, colored by life stage, with 95% confidence ellipses and intervals. (**d**) Cumulative total variance explained by the first ten PCs and variance of PC scores explained by life stage.

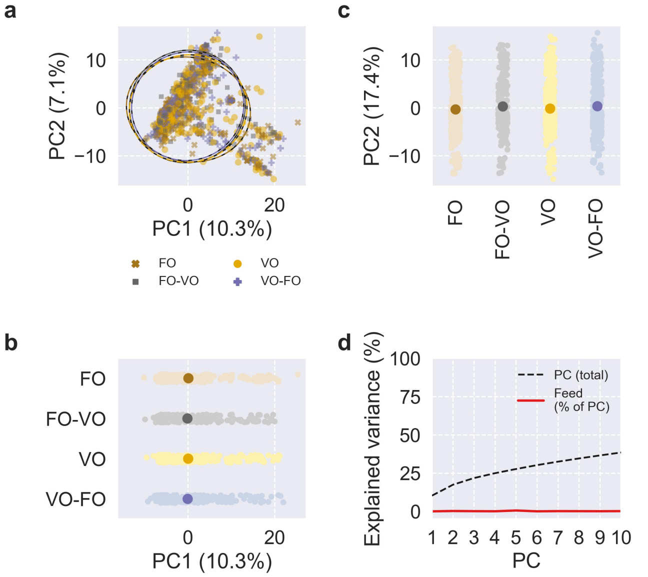

Supplementary Figure 13. Scores and explained variance from PCA of pFBA fluxes. (**a–c**) Scores of the first two PCs, colored by feed, with 95% confidence ellipses and intervals. (**d**) Cumulative total variance explained by the first ten PCs and variance of PC scores explained by feed.

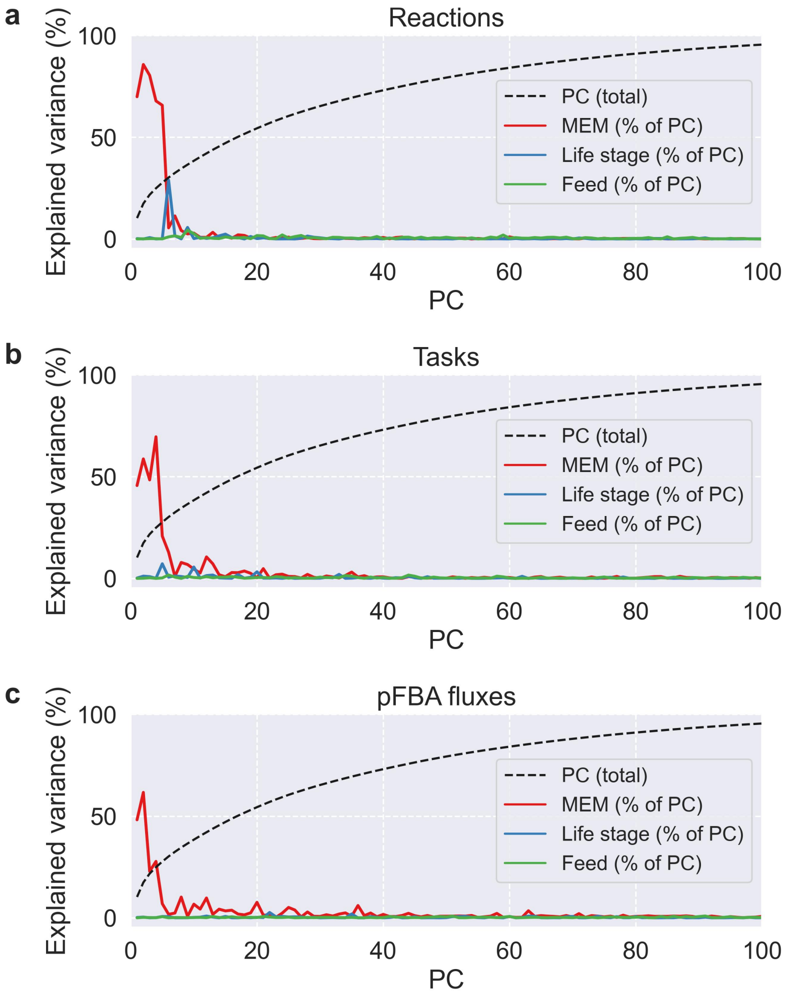

Supplementary Figure 14. Cumulative total variance explained by the first 100 PCs and variance of PC scores explained by MEM, life stage, and feed from PCA of reaction presence, task feasibility, and pFBA fluxes.

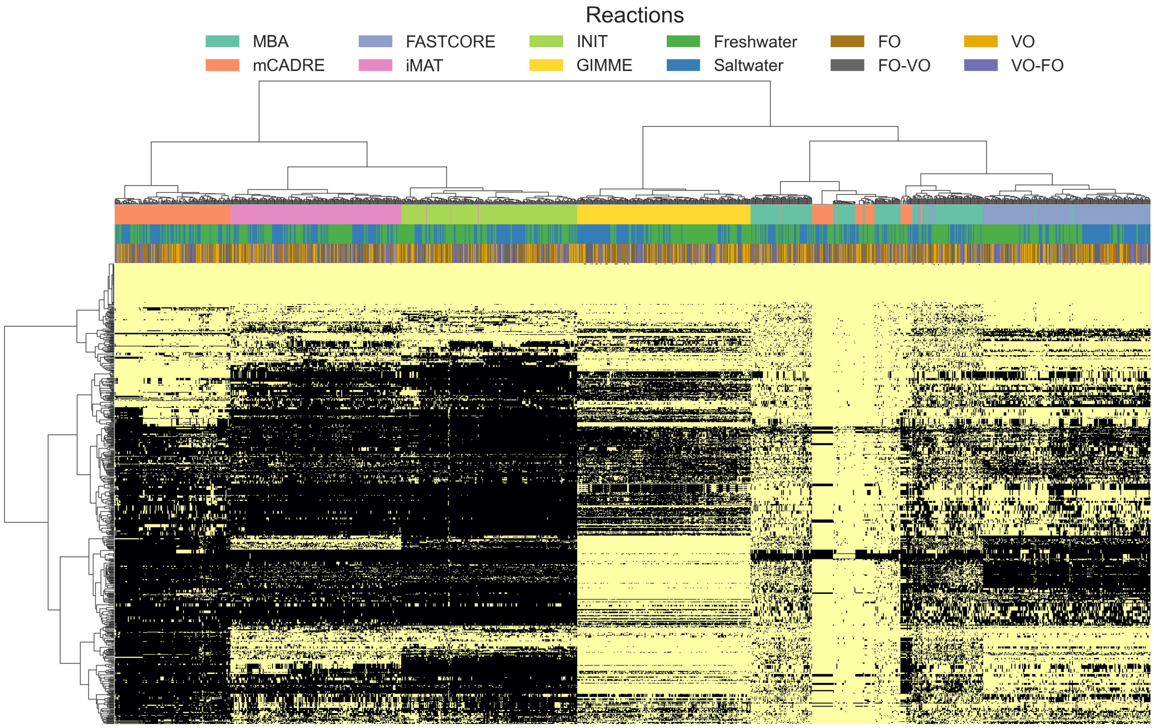

Supplementary Figure 15. Clustered heatmap of reaction presence. Rows are reactions, columns are models (samples), and a yellow cell indicates that a reaction is present in a model. Rows and columns are clustered by Ward's minimum variance method and columns are colored by MEM, life stage, and diet.

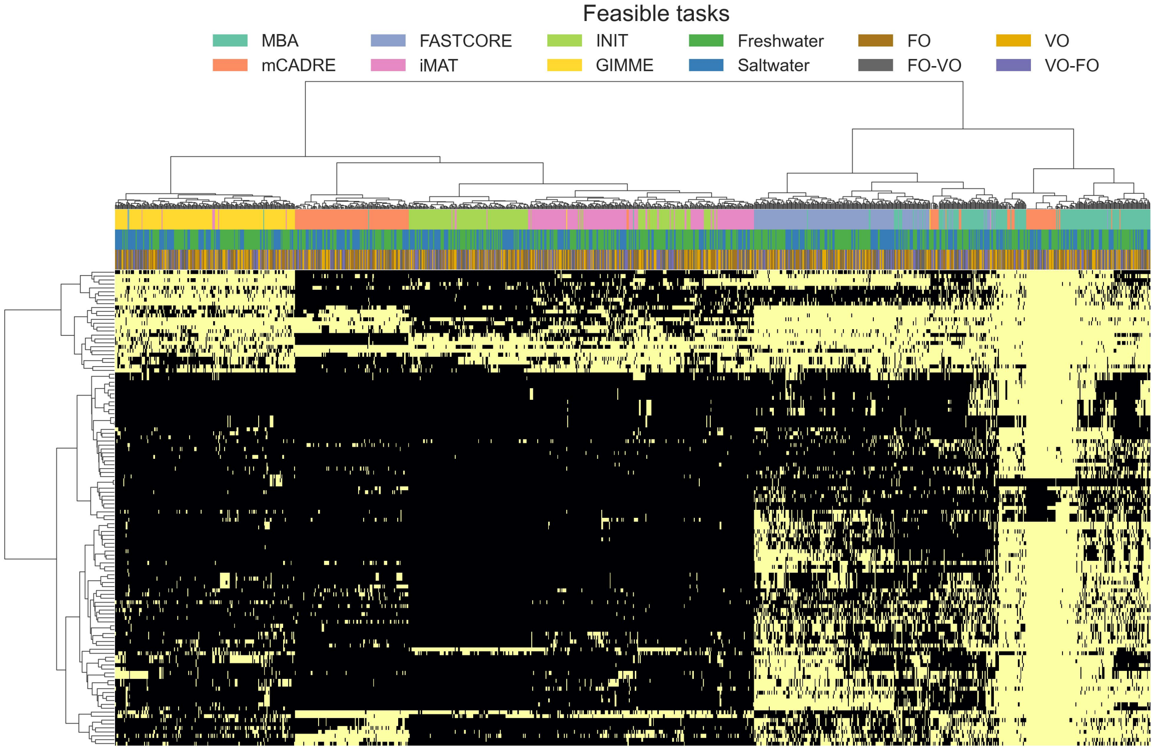

Supplementary Figure 16. Clustered heatmap of task feasibility. Rows are metabolic tasks, columns are models (samples), and a yellow cell indicates that a task can be performed by a model. Rows and columns are clustered by Ward's minimum variance method and columns are colored by MEM, life stage, and diet.

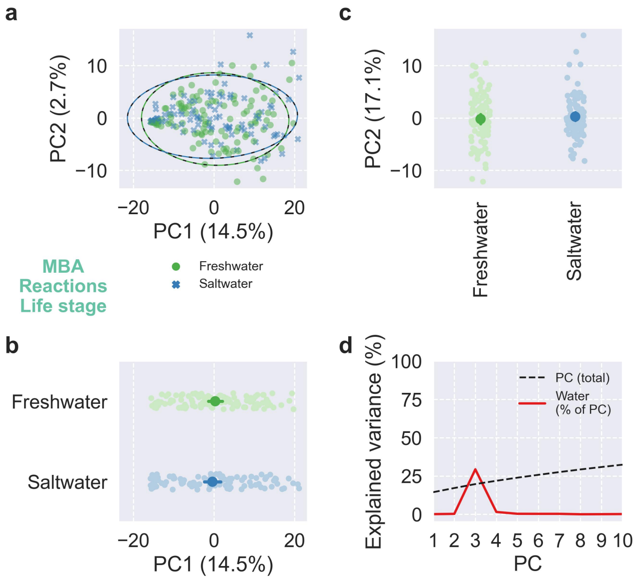

Supplementary Figure 17. Scores and explained variance from PCA of reaction presence for MBA. (**a–c**) Scores of the first two PCs, colored by life stage, with 95% confidence ellipses and intervals. (**d**) Cumulative total variance explained by the first ten PCs and variance of PC scores explained by life stage.

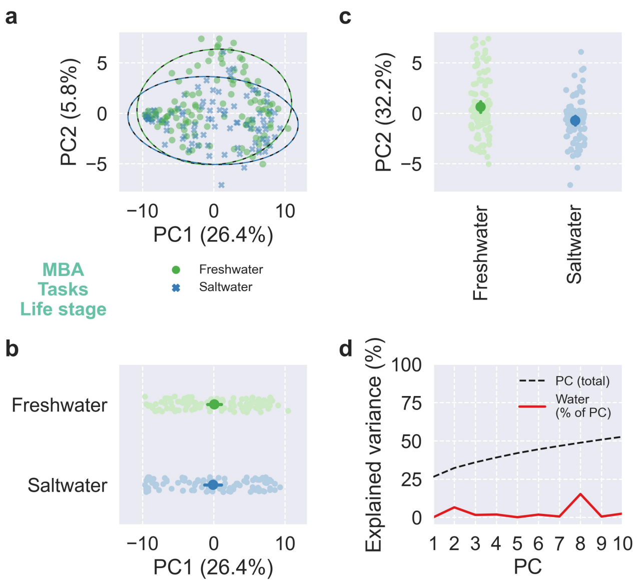

Supplementary Figure 18. Scores and explained variance from PCA of task feasibility for MBA. (**a–c**) Scores of the first two PCs, colored by life stage, with 95% confidence ellipses and intervals. (**d**) Cumulative total variance explained by the first ten PCs and variance of PC scores explained by life stage.

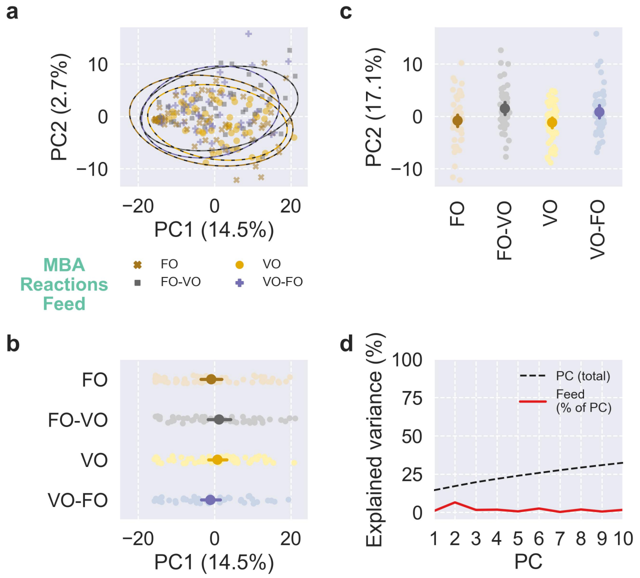

Supplementary Figure 19. Scores and explained variance from PCA of reaction presence for MBA. (**a–c**) Scores of the first two PCs, colored by feed, with 95% confidence ellipses and intervals. (**d**) Cumulative total variance explained by the first ten PCs and variance of PC scores explained by feed.

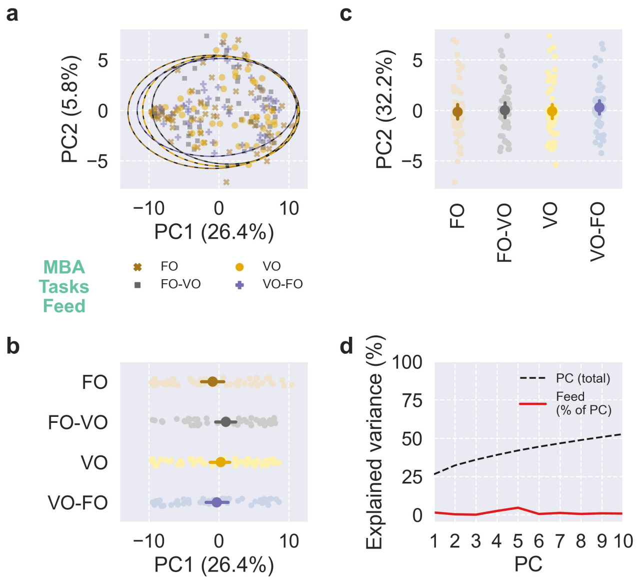

Supplementary Figure 20. Scores and explained variance from PCA of task feasibility for MBA. (**a–c**) Scores of the first two PCs, colored by feed, with 95% confidence ellipses and intervals. (**d**) Cumulative total variance explained by the first ten PCs and variance of PC scores explained by feed.

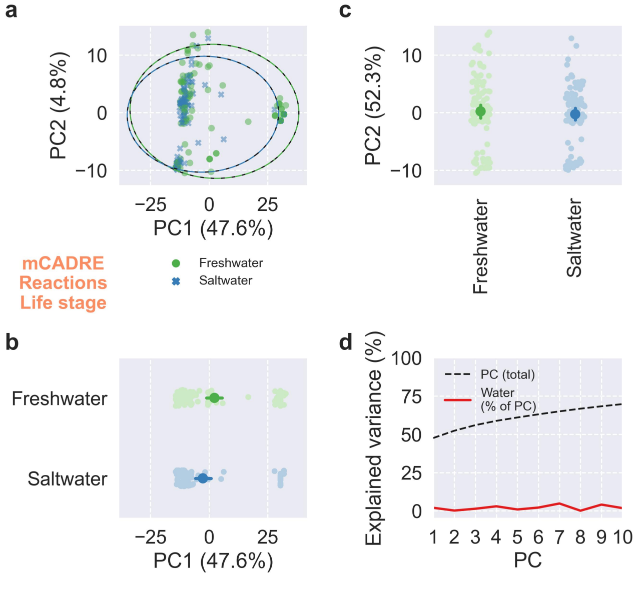

Supplementary Figure 21. Scores and explained variance from PCA of reaction presence for mCADRE. (**a–c**) Scores of the first two PCs, colored by life stage, with 95% confidence ellipses and intervals. (**d**) Cumulative total variance explained by the first ten PCs and variance of PC scores explained by life stage.

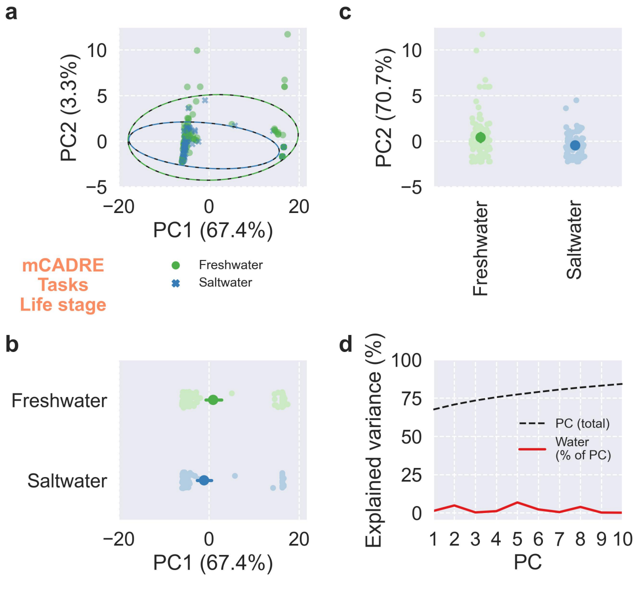

Supplementary Figure 22. Scores and explained variance from PCA of task feasibility for mCADRE. (**a–c**) Scores of the first two PCs, colored by life stage, with 95% confidence ellipses and intervals. (**d**) Cumulative total variance explained by the first ten PCs and variance of PC scores explained by life stage.

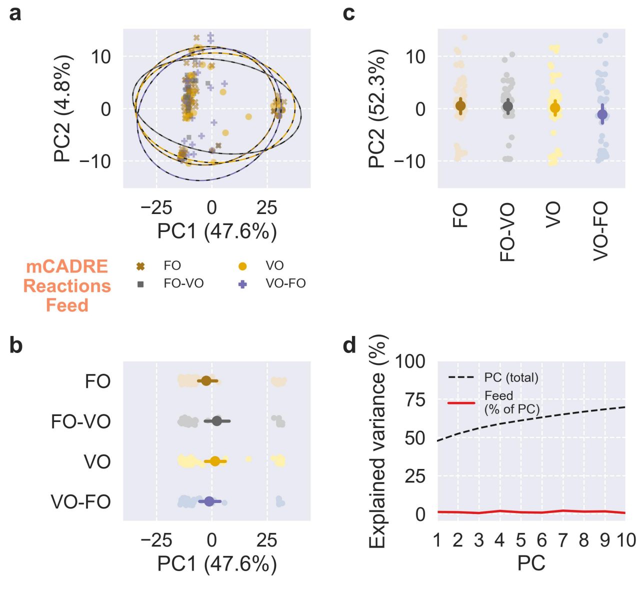

Supplementary Figure 23. Scores and explained variance from PCA of reaction presence for mCADRE. (**a–c**) Scores of the first two PCs, colored by feed, with 95% confidence ellipses and intervals. (**d**) Cumulative total variance explained by the first ten PCs and variance of PC scores explained by feed.

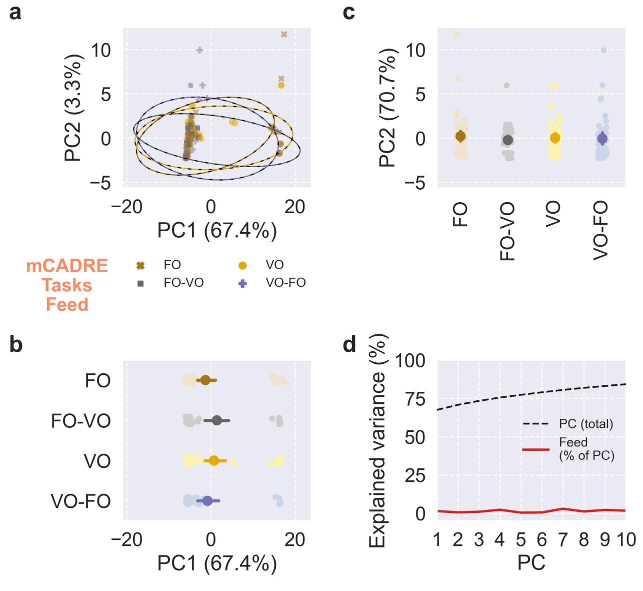

Supplementary Figure 24. Scores and explained variance from PCA of task feasibility for mCADRE. (**a–c**) Scores of the first two PCs, colored by feed, with 95% confidence ellipses and intervals. (**d**) Cumulative total variance explained by the first ten PCs and variance of PC scores explained by feed.

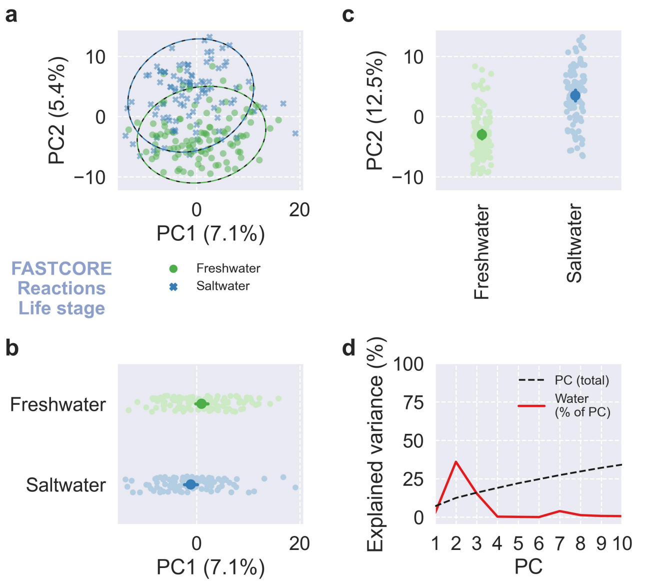

Supplementary Figure 25. Scores and explained variance from PCA of reaction presence for FASTCORE. (**a–c**) Scores of the first two PCs, colored by life stage, with 95% confidence ellipses and intervals. (**d**) Cumulative total variance explained by the first ten PCs and variance of PC scores explained by life stage.

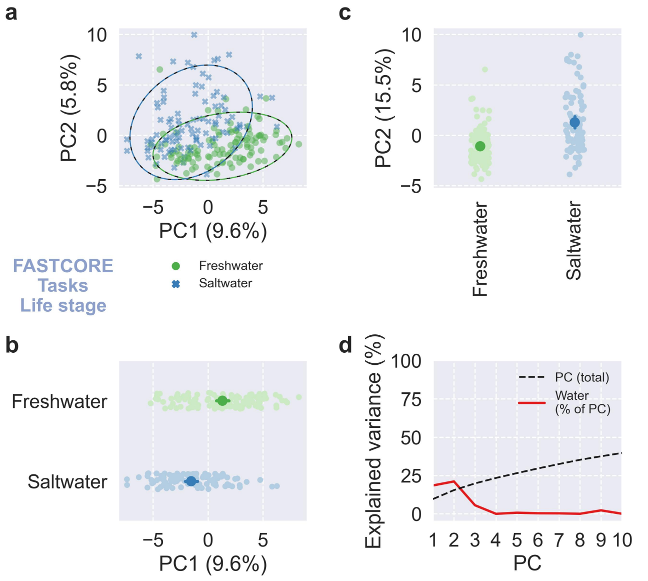

Supplementary Figure 26. Scores and explained variance from PCA of task feasibility for FASTCORE. (**a–c**) Scores of the first two PCs, colored by life stage, with 95% confidence ellipses and intervals. (**d**) Cumulative total variance explained by the first ten PCs and variance of PC scores explained by life stage.

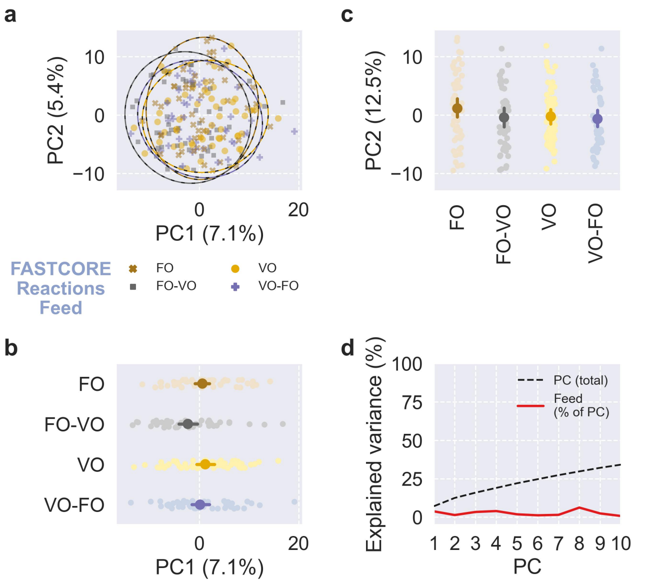

Supplementary Figure 27. Scores and explained variance from PCA of reaction presence for FASTCORE. (**a–c**) Scores of the first two PCs, colored by feed, with 95% confidence ellipses and intervals. (**d**) Cumulative total variance explained by the first ten PCs and variance of PC scores explained by feed.

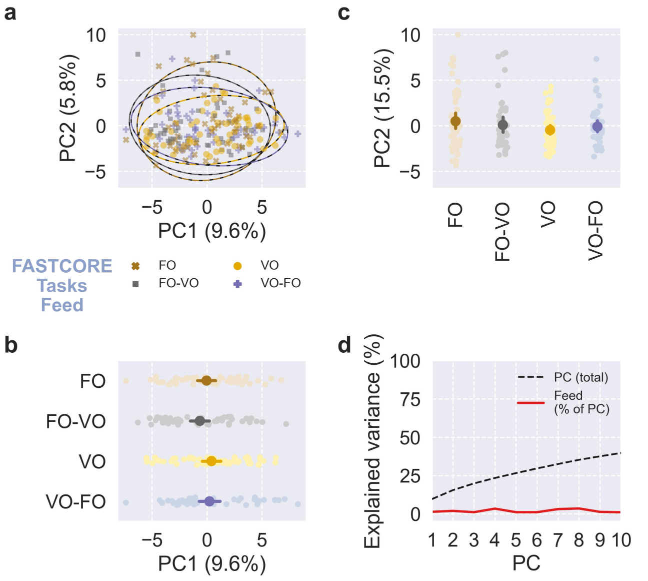

Supplementary Figure 28. Scores and explained variance from PCA of task feasibility for FASTCORE. (**a–c**) Scores of the first two PCs, colored by feed, with 95% confidence ellipses and intervals. (**d**) Cumulative total variance explained by the first ten PCs and variance of PC scores explained by feed.

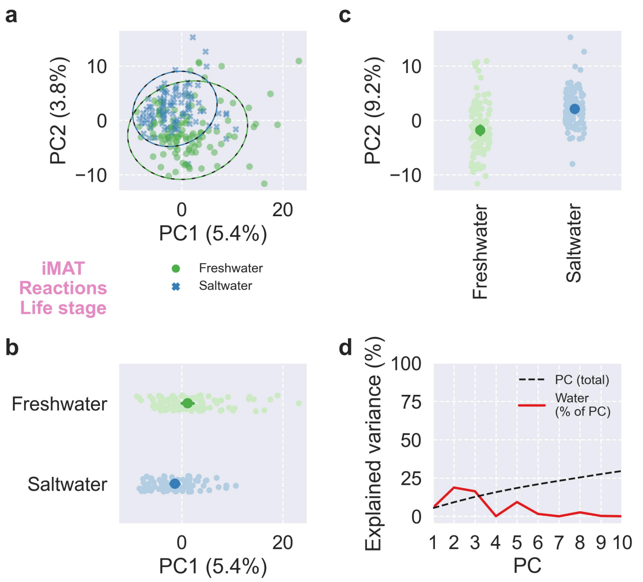

Supplementary Figure 29. Scores and explained variance from PCA of reaction presence for iMAT. (**a–c**) Scores of the first two PCs, colored by life stage, with 95% confidence ellipses and intervals. (**d**) Cumulative total variance explained by the first ten PCs and variance of PC scores explained by life stage.

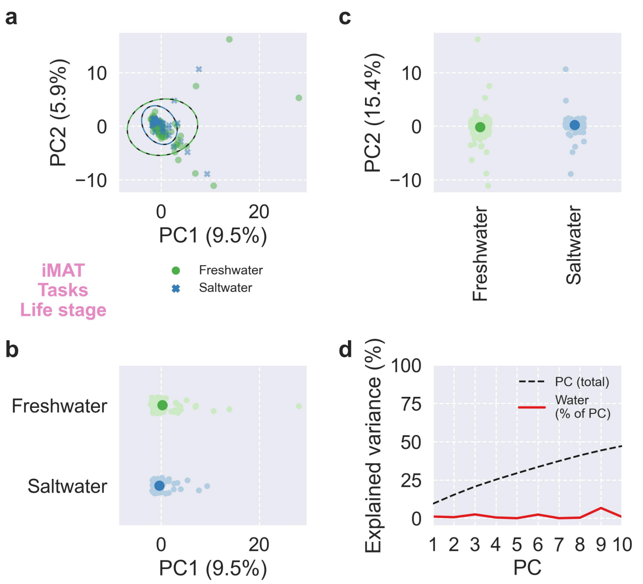

Supplementary Figure 30. Scores and explained variance from PCA of task feasibility for iMAT. (**a–c**) Scores of the first two PCs, colored by life stage, with 95% confidence ellipses and intervals. (**d**) Cumulative total variance explained by the first ten PCs and variance of PC scores explained by life stage.

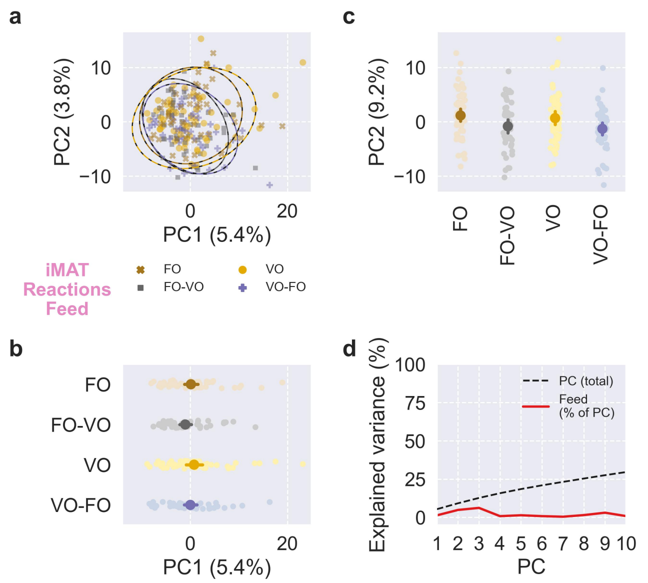

Supplementary Figure 31. Scores and explained variance from PCA of reaction presence for iMAT. (**a–c**) Scores of the first two PCs, colored by feed, with 95% confidence ellipses and intervals. (**d**) Cumulative total variance explained by the first ten PCs and variance of PC scores explained by feed.

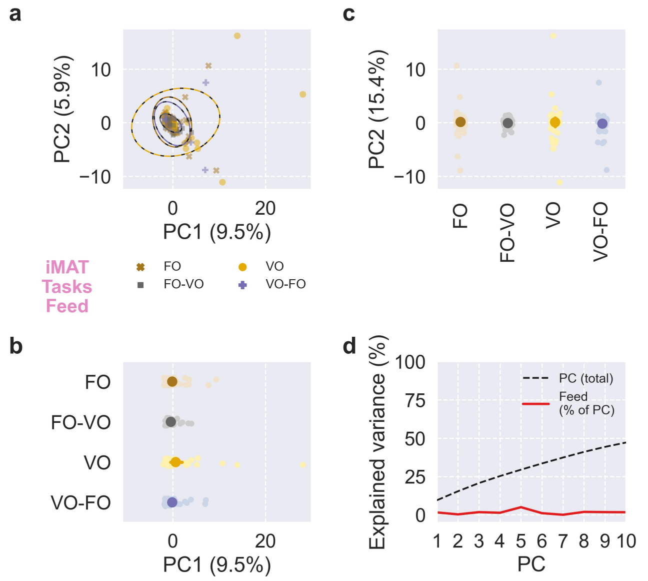

Supplementary Figure 32. Scores and explained variance from PCA of task feasibility for iMAT. (**a–c**) Scores of the first two PCs, colored by feed, with 95% confidence ellipses and intervals. (**d**) Cumulative total variance explained by the first ten PCs and variance of PC scores explained by feed.

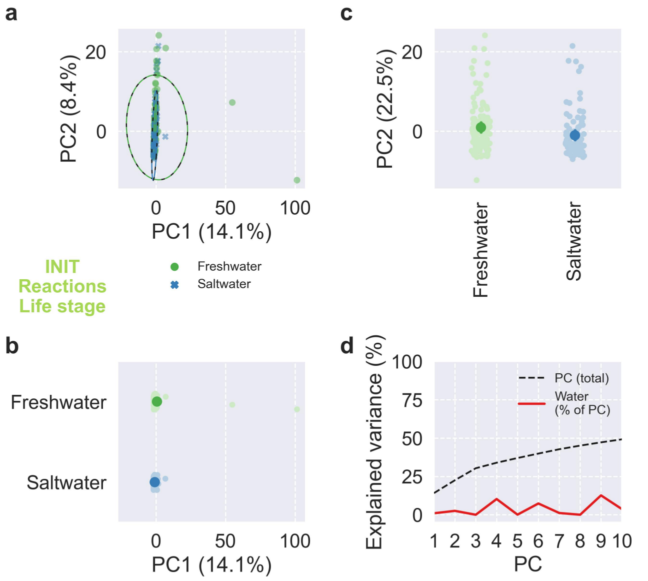

Supplementary Figure 33. Scores and explained variance from PCA of reaction presence for INIT. (**a–c**) Scores of the first two PCs, colored by life stage, with 95% confidence ellipses and intervals. (**d**) Cumulative total variance explained by the first ten PCs and variance of PC scores explained by life stage.

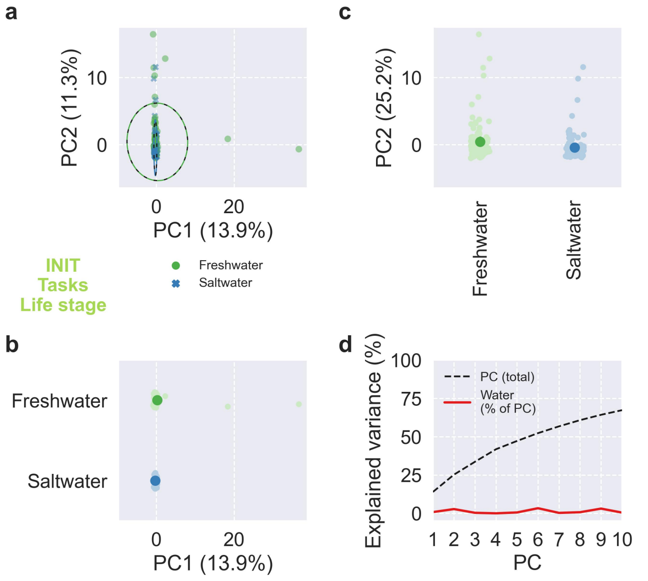

Supplementary Figure 34. Scores and explained variance from PCA of task feasibility for INIT. (**a–c**) Scores of the first two PCs, colored by life stage, with 95% confidence ellipses and intervals. (**d**) Cumulative total variance explained by the first ten PCs and variance of PC scores explained by life stage.

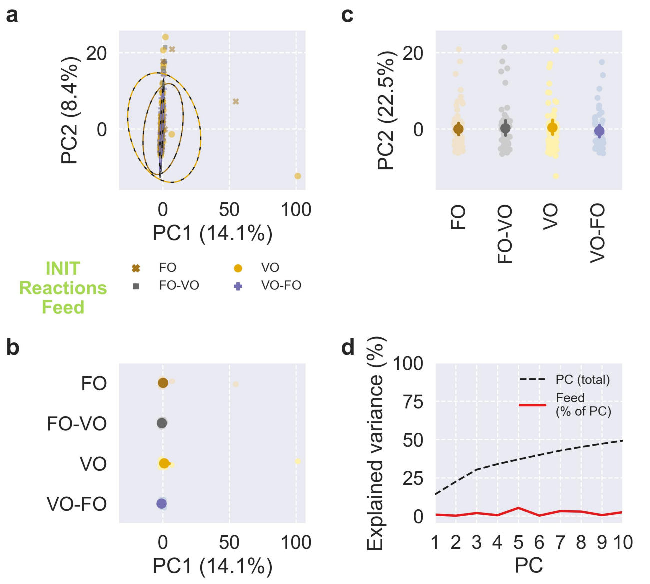

Supplementary Figure 35. Scores and explained variance from PCA of reaction presence for INIT. (**a–c**) Scores of the first two PCs, colored by feed, with 95% confidence ellipses and intervals. (**d**) Cumulative total variance explained by the first ten PCs and variance of PC scores explained by feed.

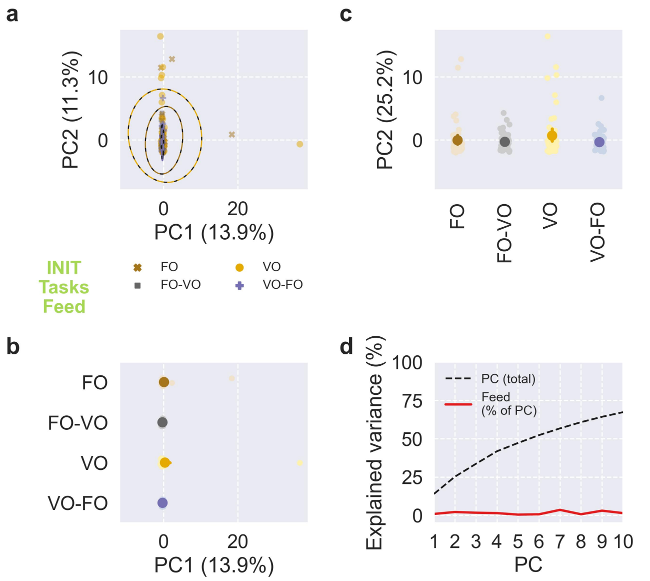

Supplementary Figure 36. Scores and explained variance from PCA of task feasibility for INIT. (**a–c**) Scores of the first two PCs, colored by feed, with 95% confidence ellipses and intervals. (**d**) Cumulative total variance explained by the first ten PCs and variance of PC scores explained by feed.

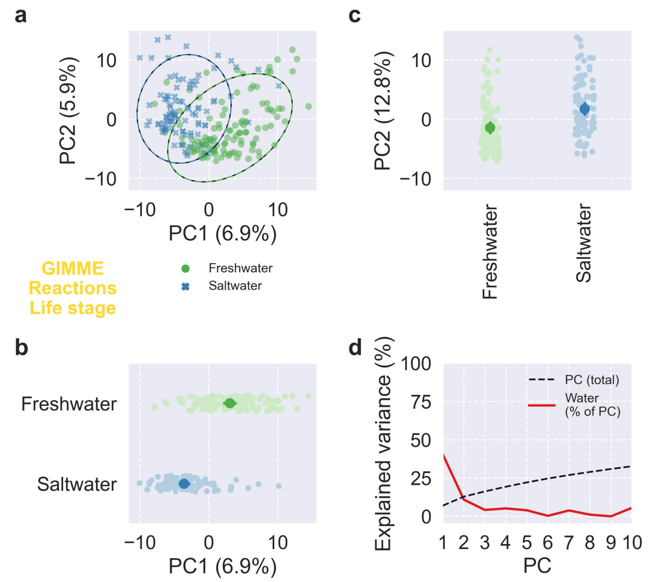

Supplementary Figure 37. Scores and explained variance from PCA of reaction presence for GIMME. (**a–c**) Scores of the first two PCs, colored by life stage, with 95% confidence ellipses and intervals. (**d**) Cumulative total variance explained by the first ten PCs and variance of PC scores explained by life stage.

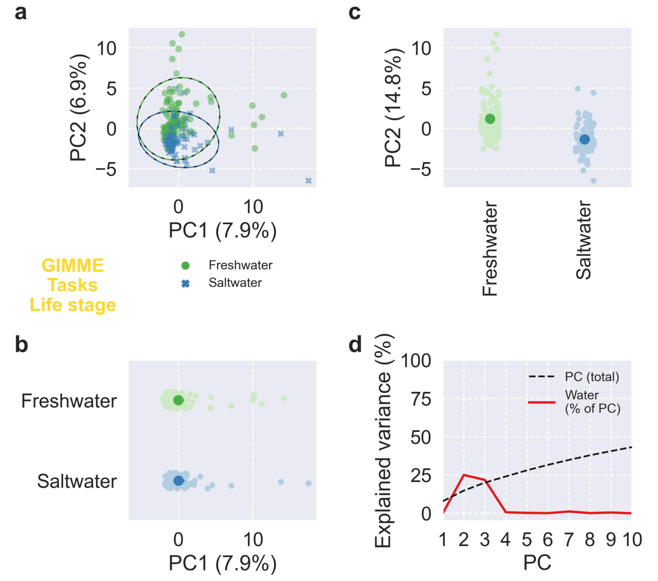

Supplementary Figure 38. Scores and explained variance from PCA of task feasibility for GIMME. (**a–c**) Scores of the first two PCs, colored by life stage, with 95% confidence ellipses and intervals. (**d**) Cumulative total variance explained by the first ten PCs and variance of PC scores explained by life stage.

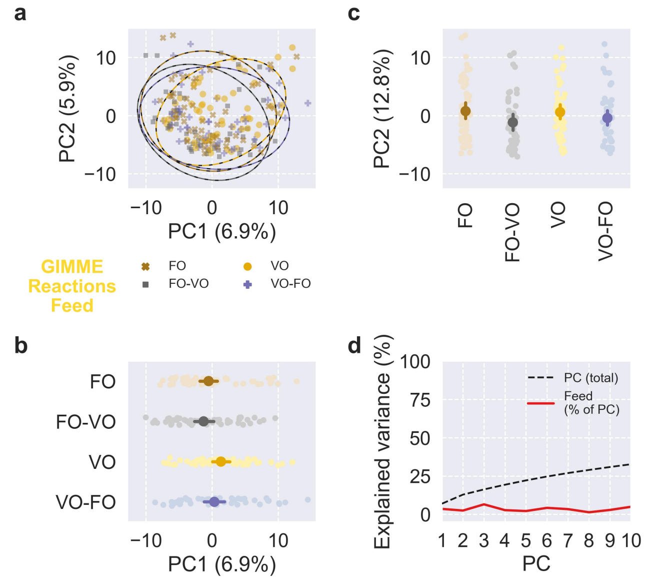

Supplementary Figure 39. Scores and explained variance from PCA of reaction presence for GIMME. (**a–c**) Scores of the first two PCs, colored by feed, with 95% confidence ellipses and intervals. (**d**) Cumulative total variance explained by the first ten PCs and variance of PC scores explained by feed.

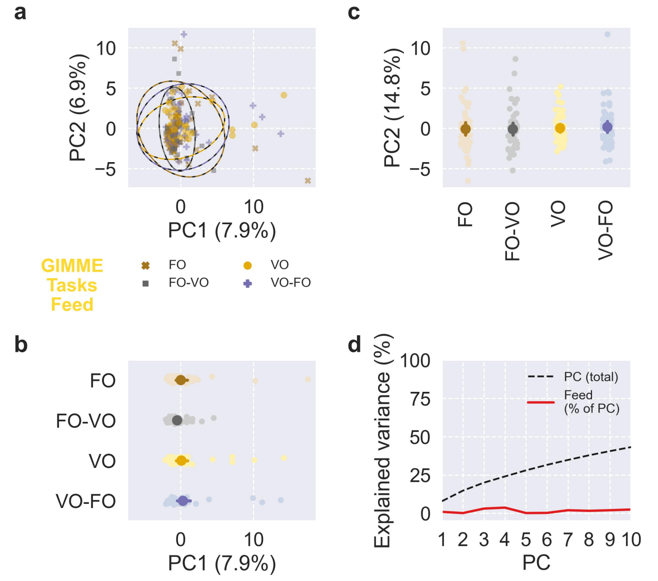

Supplementary Figure 40. Scores and explained variance from PCA of task feasibility for GIMME. (**a–c**) Scores of the first two PCs, colored by feed, with 95% confidence ellipses and intervals. (**d**) Cumulative total variance explained by the first ten PCs and variance of PC scores explained by feed.

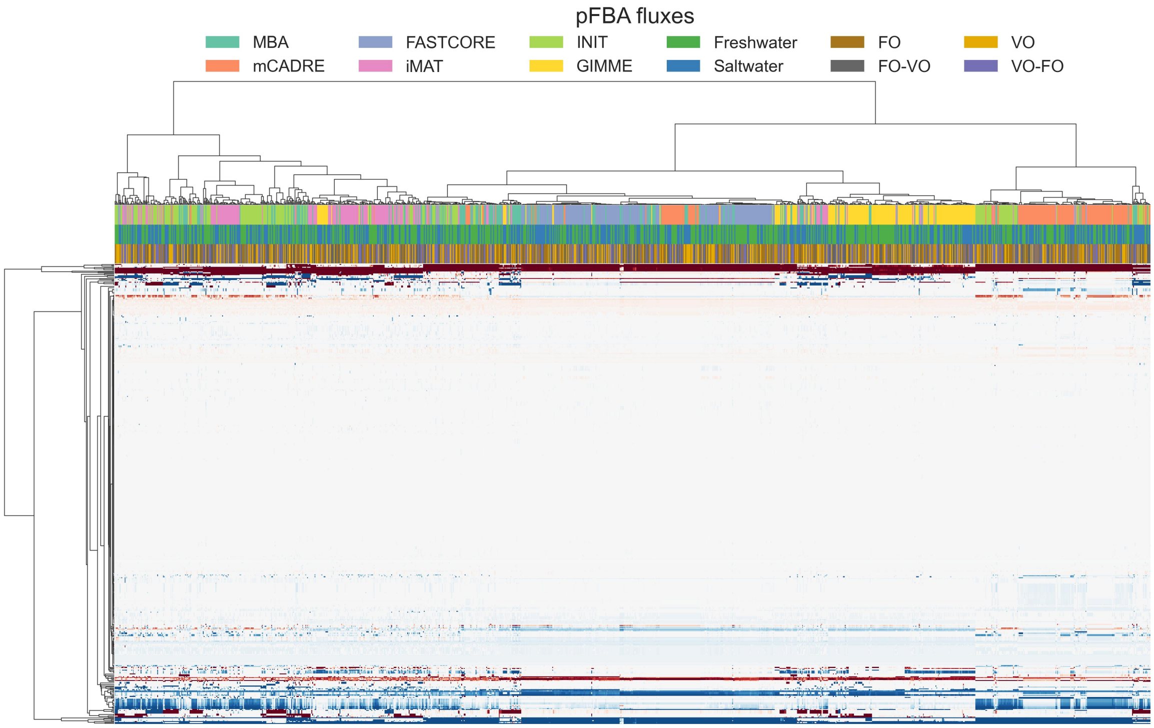

Supplementary Figure 41. Clustered heatmap of pFBA fluxes. Rows are reactions, columns are models (samples), and each cell indicates the pFBA flux of a reaction in a model. Colors indicate negative (red) and positive (blue) flux. Rows and columns are clustered by Ward's minimum variance method and columns are colored by MEM, life stage, and diet.

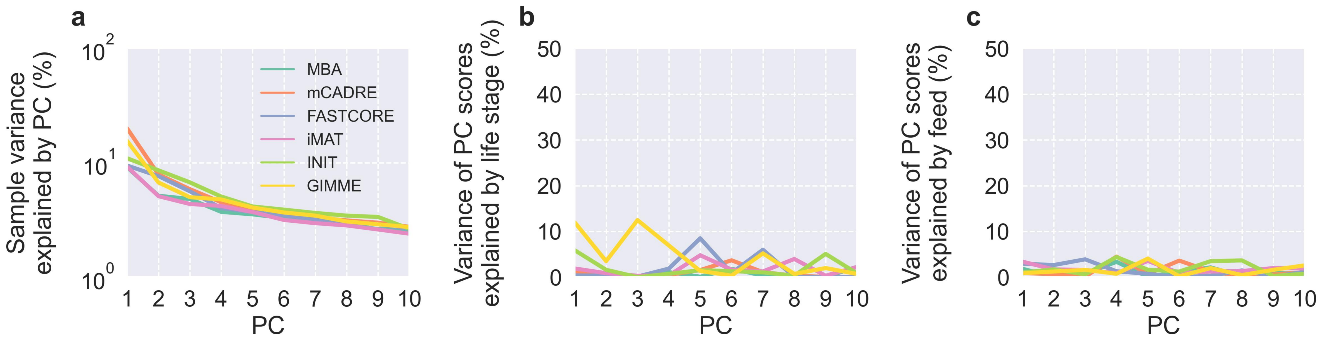

Supplementary Figure 42. Results from PCA of pFBA fluxes performed separately for each MEM. Sample variance explained by each PC and variance of PC scores explained by life stage and feed are shown for the first ten PCs.

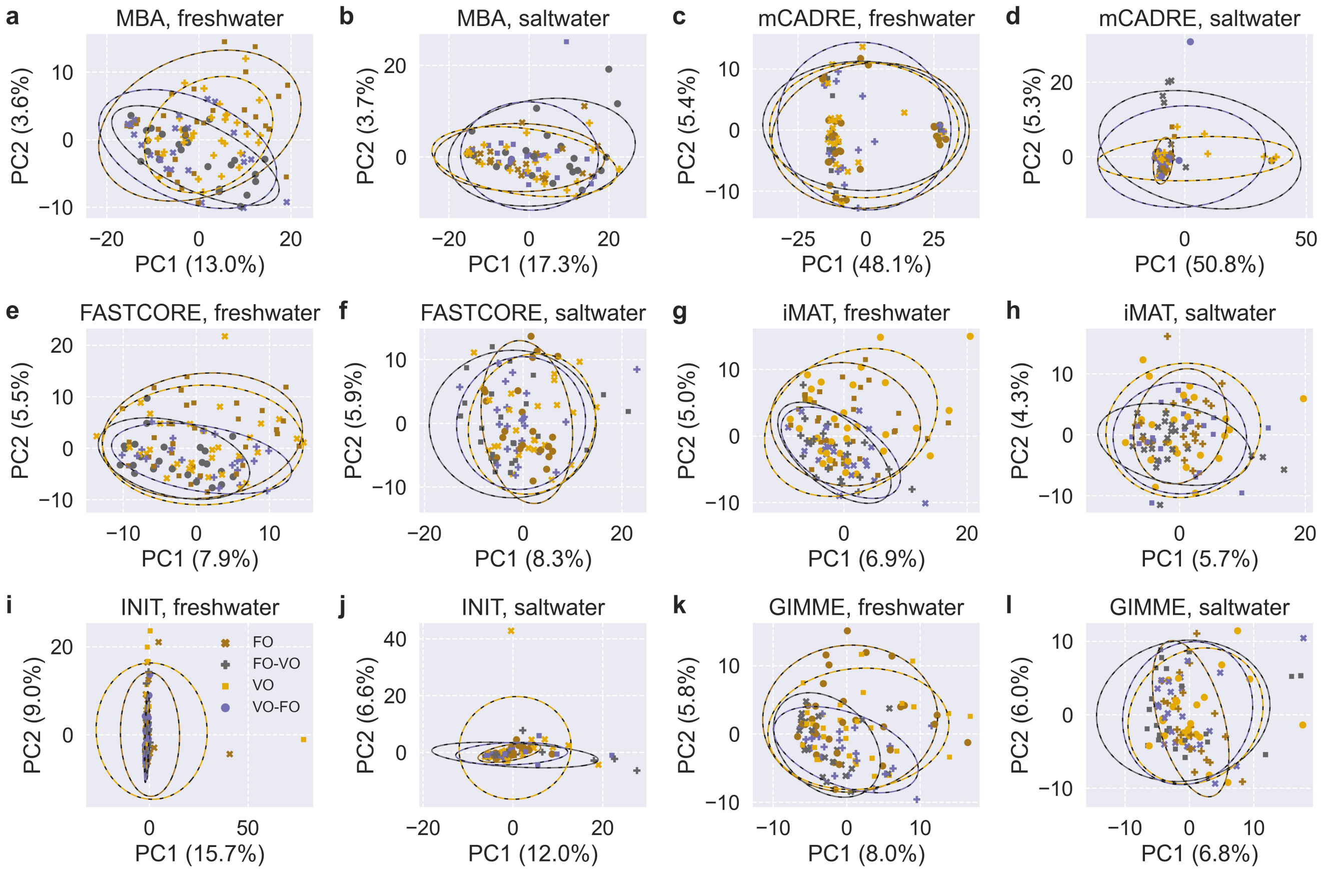

Supplementary Figure 43. PCA of reaction presence within MEMs and life stages. Scores and explained variance of the first two PCs are shown, with colors indicating feeds and 95% confidence ellipses.

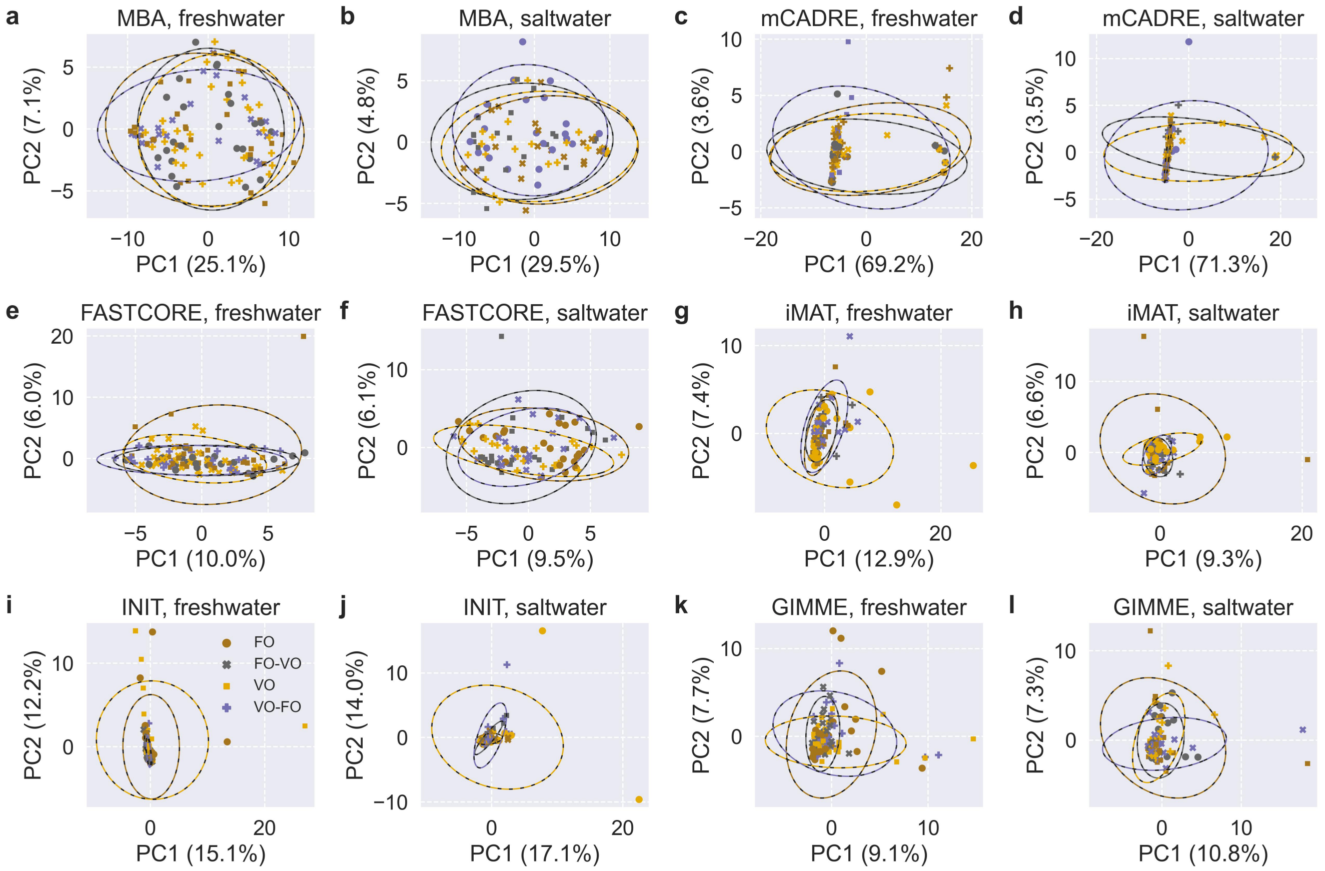

Supplementary Figure 44. PCA of task feasibility within MEMs and life stages. Scores and explained variance of the first two PCs are shown, with colors indicating feeds and 95% confidence ellipses.

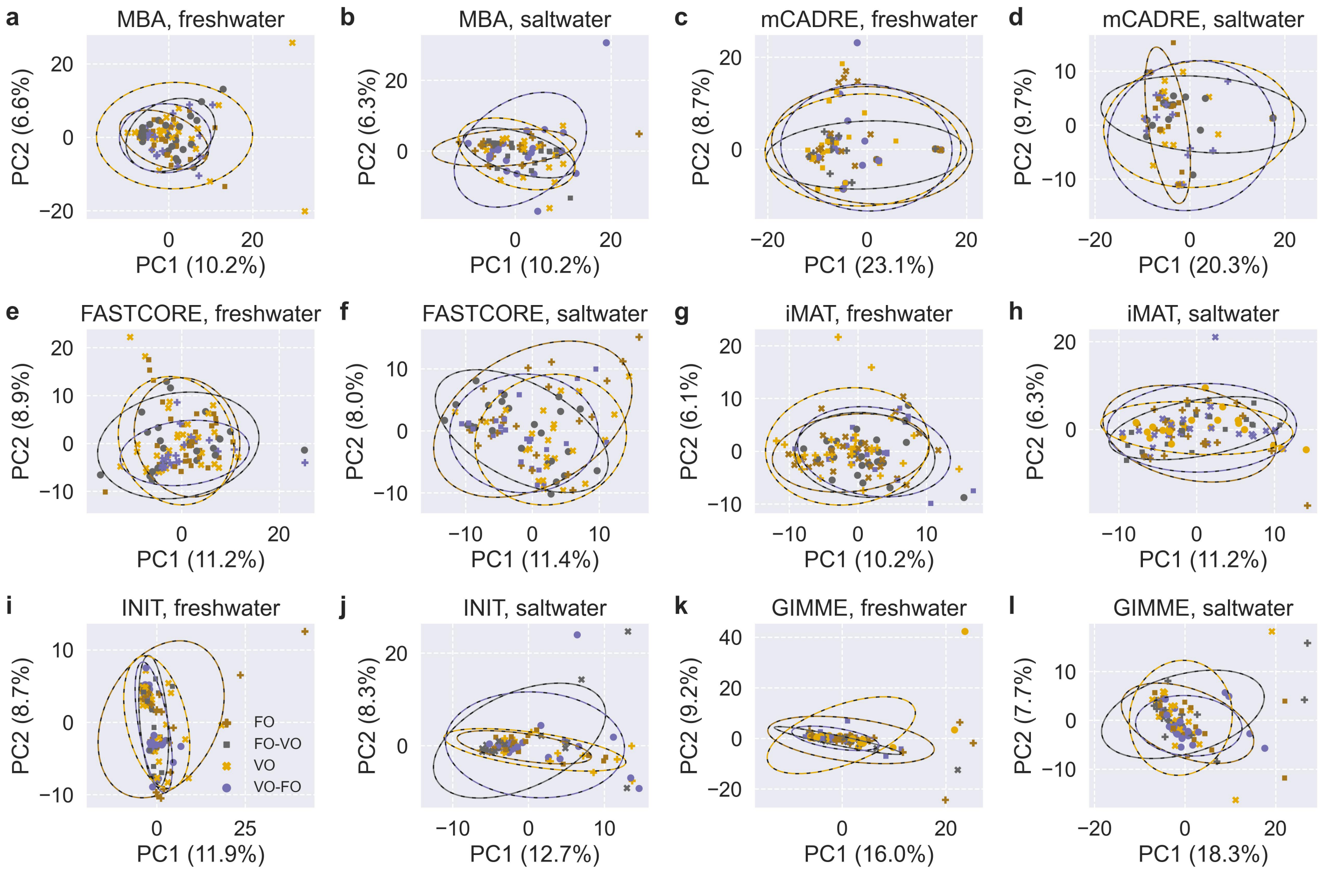

Supplementary Figure 45. PCA of pFBA fluxes within MEMs and life stages. Scores and explained variance of the first two PCs are shown, with colors indicating feeds and 95% confidence ellipses.

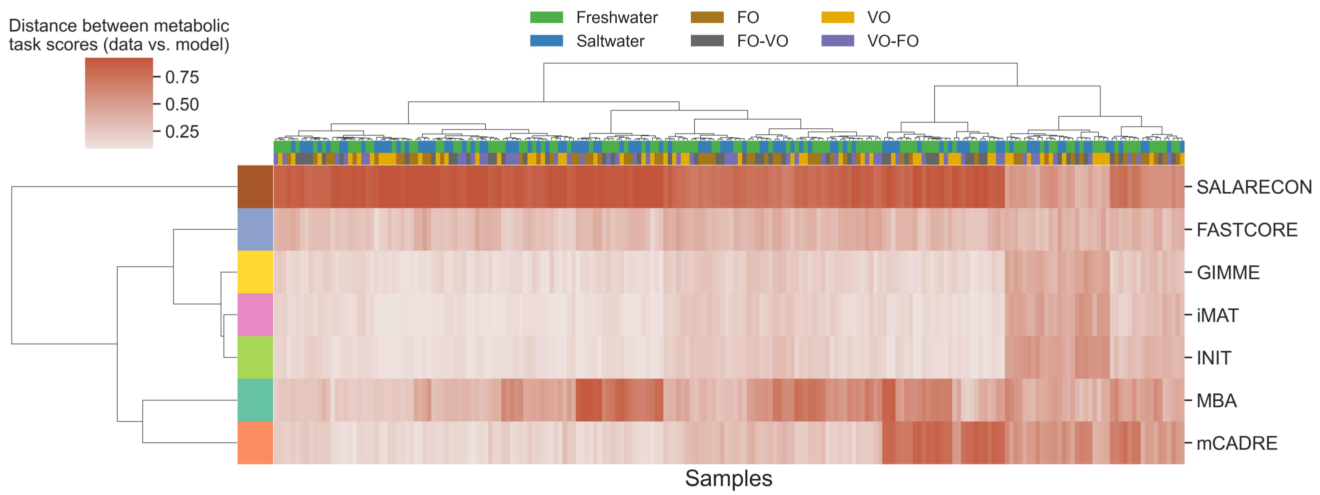

Supplementary Figure 46. Clustered heatmap of metabolic task distances. Rows are MEMs, columns are models, and cells indicate the Normalized Hamming distance between task scores inferred from data and task scores predicted by models. Rows and columns are clustered by Ward's minimum variance method, rows are colored by MEM, and columns are colored by life stage and diet.
